# MGT–UNet: a hybrid CNN–transformer network with multi-scale feature learning and global context modeling for breast ultrasound segmentation

**DOI:** 10.1186/s12880-026-02687-4

**Published:** 2026-08-17

**Authors:** Hao Vu Le, Hieu Trung Huynh

**Affiliations:** Faculty of Information Technology, Industrial University of Ho Chi Minh City, No. 12 Nguyen Van Bao, Hanh Thong Ward, Ho Chi Minh City, 700000 Vietnam

**Keywords:** Breast ultrasound, Medical image segmentation, Hybrid CNN–Transformer, Multi-scale feature learning, Global context modeling

## Abstract

**Background:**

Accurate breast ultrasound image segmentation remains challenging because speckle noise, low contrast, and heterogeneous lesion appearance often degrade lesion boundary delineation. Convolutional neural networks provide effective local feature extraction but have limited capability for modeling long-range contextual information. Although transformer-based architectures improve global context modeling, effectively combining local multi-scale representations with global contextual information remains challenging.

**Methods:**

We propose MGT–UNet, a hybrid CNN–Transformer segmentation network that integrates multi-scale feature learning with global context modeling for breast ultrasound image segmentation. The encoder employs a Tri-Scale Context Extractor to learn hierarchical multi-scale representations, while a Global Context Transformer models long-range contextual dependencies at the bottleneck. The network was evaluated primarily on the BUSI breast ultrasound dataset using Dice coefficient, Intersection-over-Union, Hausdorff Distance at the 95th percentile, Precision, and Recall. Supplementary evaluations were conducted on the ISIC2018 dermoscopic dataset and a binary variant of the Synapse computed tomography dataset using the same segmentation protocol. To ensure reliability, performance metrics were averaged over multiple independent training runs with different random seeds.

**Results:**

On the BUSI dataset, MGT–UNet achieved the highest Dice coefficient of 77.74%, outperforming representative CNN-, Transformer-, and hybrid segmentation models while also achieving the lowest HD$_{95}$. The results indicate improved segmentation performance on the BUSI dataset, which contains images affected by speckle noise and weak boundary contrast. Evaluations on dermoscopic and computed tomography datasets complement these findings by examining the behavior of the proposed architecture across diverse modalities.

**Conclusions:**

Our results suggest that combining multi-scale feature learning with transformer-based global context modeling is beneficial for breast ultrasound segmentation. The proposed architecture provides a basis for further investigation of hybrid CNN–Transformer models in medical image segmentation.

## Introduction

Accurate medical image segmentation plays a fundamental role in modern computer-assisted diagnosis and clinical decision support systems. Precise delineation of anatomical structures and pathological regions enables quantitative analysis of disease progression, treatment planning, and outcome monitoring across a wide range of medical imaging modalities. In recent years, deep learning-based segmentation methods have substantially improved the automation, accuracy, and reliability of medical image analysis across diverse biomedical imaging applications. Among these applications, breast lesion segmentation in ultrasound imaging is particularly important for early breast cancer detection and screening programs.

Breast ultrasound imaging is widely used as a complementary diagnostic modality due to its safety, accessibility, and cost-effectiveness [1, 2]. However, automated segmentation remains challenging because ultrasound images often suffer from speckle noise, low contrast, and imaging artifacts [1, 3]. Furthermore, variations in lesion appearance, size, and location make accurate delineation even more challenging [2]. To accurately delineate breast lesions in noisy images, segmentation models must effectively combine fine-grained spatial details with robust contextual information.

Accordingly, this study focuses on breast ultrasound segmentation using the BUSI dataset [4], a representative benchmark for evaluating models under challenging imaging conditions. To further examine the behavior of the proposed architecture, we conducted additional experiments on the ISIC2018 dermoscopic lesion dataset [5, 6] and a binary variant of the Synapse multi-organ computed tomography (CT) dataset (hereafter referred to as Synapse) [7] using the same experimental protocol. Although the original Synapse dataset is a multi-class, multi-organ benchmark, the binary version used in this study enables a consistent binary segmentation protocol across all evaluated datasets. This additional evaluation complements the primary BUSI findings by illustrating how the proposed architecture performs on a different imaging modality.

Deep learning has substantially advanced medical image segmentation, primarily through encoder–decoder architectures like U-Net and its variants. By leveraging skip connections, these networks achieve effective hierarchical feature representation and precise spatial localization [8–10]. However, conventional convolutional neural networks (CNNs) rely on localized receptive fields, limiting their capacity to model long-range contextual dependencies [11, 12]. To overcome this constraint, transformer-based models employ self-attention mechanisms to capture global context [13–16]. More recently, hybrid CNN–Transformer architectures, such as TransFuse [17], UTNet [18], MISSFormer [19], and BACFormer [20], have emerged to combine the complementary strengths of local convolutional feature extraction and global transformer-based modeling.

Despite recent progress, effectively integrating multi-scale local feature representations with long-range contextual modeling remains a key challenge in hybrid CNN–Transformer architectures. This limitation is particularly important for breast ultrasound segmentation, where accurate lesion delineation requires both fine local detail and robust global semantic understanding.

To address this challenge, we propose MGT–UNet, a hybrid CNN–Transformer architecture that combines a Tri-Scale Context Extractor (TCE) for hierarchical multi-scale feature learning with a Global Context Transformer (GCT) for bottleneck-level contextual reasoning. Together, these modules provide complementary local and global representations within a unified encoder–decoder framework. The main contributions of this work are summarized as follows:

1. We propose MGT–UNet, a hybrid CNN–Transformer architecture for breast ultrasound segmentation that integrates a TCE and a GCT.
2. The proposed TCE captures complementary local representations across multiple receptive fields, while the GCT models long-range contextual dependencies using transformer self-attention.
3. We conduct comprehensive experiments to demonstrate the effectiveness of the proposed architecture on the BUSI breast ultrasound benchmark. Furthermore, evaluations on the ISIC2018 dermoscopic dataset and the Synapse dataset provide additional evaluation using a consistent binary segmentation protocol.
4. Ablation studies show that the TCE and GCT provide complementary performance gains, supporting the benefit of coupling multi-scale feature learning with global contextual modeling for breast ultrasound segmentation.

## Related work

### Encoder–decoder architectures for medical image segmentation

Deep convolutional encoder–decoder architectures form the backbone of modern medical image segmentation. Fully Convolutional Networks (FCNs) first demonstrated that dense prediction tasks can be addressed through end-to-end convolutional learning, bypassing fully connected layers to achieve efficient pixel-wise prediction across entire images [21]. Building upon this paradigm, U-Net introduced a symmetric structure with skip connections that transfer high-resolution spatial details directly from the encoder to the decoder, facilitating precise localization in biomedical tasks [8]. Owing to its robust performance and computational efficiency, U-Net has served as the baseline for numerous subsequent medical imaging architectures.

Following the success of U-Net, numerous extensions have emerged to optimize representation learning, feature reuse, and training stability. V-Net extended encoder–decoder architectures to volumetric data by incorporating residual connections and fully three-dimensional convolutions [22]. Concurrently, DenseUNet variants, such as H-DenseUNet, utilize dense connectivity to maximize feature reuse and facilitate information flow across layers [10]. To stabilize optimization in deeper networks, residual-based encoder–decoder designs leverage identity mappings that mitigate gradient degradation [23]. Beyond structural re-engineering, attention mechanisms have become pivotal. For instance, ResUNet++ integrates residual units, squeeze-and-excitation blocks, and atrous spatial pyramid pooling with attention modules to enhance semantic segmentation performance [24]. More recently, lightweight attention modules have been embedded into encoder–decoder frameworks to refine feature maps. Representative paradigms include CBAM, which sequentially infers channel and spatial attention [25], and SCSE, which concurrently recalibrates channel-wise and spatial feature responses for targeted medical image segmentation [26].

Despite these architectural advances, purely convolutional networks remain constrained by their reliance on progressively expanding receptive fields to capture context. Consequently, recent efforts have turned to complementary mechanisms, such as multi-scale feature learning and transformer-based modeling, to overcome these local limitations.

### Multi-scale feature learning

Anatomical structures and pathological regions in medical images often exhibit substantial variability in size, shape, and spatial distribution [27]. This heterogeneity has motivated the development of multi-scale feature learning strategies designed to capture both fine structural details and broader context. Atrous (dilated) convolution expands the receptive field without increasing the parameter count or sacrificing spatial resolution [28]. Building upon this concept, the DeepLab framework employs Atrous Spatial Pyramid Pooling (ASPP) to aggregate features across multiple sampling rates, thereby capturing context across diverse spatial dimensions [29, 30]. These approaches demonstrate that integrating features from varied receptive fields yields a richer representation of anatomical structures subject to severe scale variation.

Beyond dilated convolutions, alternative architectures explicitly integrate information across multiple spatial resolutions. For instance, PSPNet introduces pyramid pooling to capture both global and regional context [31], whereas Feature Pyramid Networks (FPNs) combine semantically rich deep features with spatially precise shallow details through a top-down hierarchy [32]. HRNet maintains high-resolution representations throughout the network by repeatedly exchanging information across distinct scales [33]. Similarly, Res2Net constructs hierarchical multi-scale representations within individual residual blocks, facilitating flexible receptive-field modeling [34].

Within encoder–decoder frameworks, multi-scale feature learning has been advanced through hierarchical aggregation across multiple semantic levels. For instance, UNet++ introduces nested skip connections to progressively narrow the semantic gap between encoder and decoder features [9], whereas UNet3+ aggregates full-scale multi-resolution features during decoder reconstruction to maximize cross-scale interactions [35]. Sharp U-Net further refines multi-scale representations by incorporating depthwise convolution-based sharpening, thereby enhancing boundary preservation while maintaining computational efficiency [36].

Beyond architectural innovations, advances in medical image segmentation have also been facilitated by standardized benchmark datasets and improved learning strategies. The MoNuSAC2020 challenge established a large multi-organ nuclei segmentation and classification benchmark for objective evaluation of segmentation algorithms in computational pathology [37]. More recently, Masked Supervised Learning introduced a masked supervision strategy that improves semantic segmentation by enhancing contextual representation learning without modifying the underlying segmentation architecture [38]. These developments complement architectural advances by highlighting the importance of both rigorous benchmarking and effective training methodologies in advancing medical image segmentation.

Multi-scale feature learning is crucial for medical image segmentation due to the substantial scale variations inherent in anatomical structures. This challenge is magnified in breast ultrasound imaging, where severe speckle noise, heterogeneous lesion appearances, and poorly defined boundaries complicate precise delineation [2]. These factors motivate the development of multi-scale feature extraction modules designed to capture complementary representations across multiple receptive fields, thereby improving target delineation under noisy imaging conditions.

### Transformer architectures for medical image segmentation

Transformer models have emerged as a powerful paradigm for visual representation learning due to their capacity to capture long-range dependencies via self-attention mechanisms. The Vision Transformer (ViT) adapts this framework to image analysis by treating images as sequences of patches, facilitating global contextual modeling that extends beyond the localized receptive fields of convolutional operations [12]. Compared with conventional convolutional networks, transformers naturally encode overarching spatial relationships, making them suitable for modeling complex spatial relationships in medical image segmentation tasks.

Building upon this concept, several transformer architectures have been developed for medical image segmentation. Medical Transformer introduces axial attention to enhance context modeling in high-resolution medical images [39]. Swin-Unet adopts a hierarchical shifted-window self-attention mechanism to optimize computational efficiency while preserving global representations [15]. Similarly, UTNet integrates efficient self-attention within a U-shaped framework to balance computational overhead with contextual modeling capability [18].

Despite these advantages, transformer-based models inherently lack the strong inductive biases of convolutional networks, often necessitating larger training datasets to achieve robust generalization [12, 39]. These distinct yet complementary strengths have motivated the development of hybrid CNN–Transformer architectures that integrate localized convolutional feature extraction with transformer-based global context modeling.

### Hybrid CNN–transformer architectures

Hybrid CNN–Transformer architectures have emerged to combine the localized representation capabilities of convolutional networks with the global contextual modeling of transformers. Rather than replacing convolutions entirely, these frameworks integrate convolutional operations with self-attention mechanisms to exploit their complementary strengths. A pioneering framework in this domain is TransUNet, which couples a convolutional backbone with a transformer encoder to merge local feature extraction and global context modeling [13]. Extending this approach, TransFuse processes parallel convolutional and transformer branches to extract complementary representations prior to feature fusion [17]. For volumetric medical image segmentation, CoTr introduces transformer-based contextual interaction driven by deformable attention mechanisms [40]. Recent developments have focused on increasingly sophisticated hybrid configurations. For instance, MISSFormer employs multi-scale transformer interactions to enrich feature representations across multiple spatial resolutions [19], whereas UCTransNet bridges encoder–decoder communication via channel-wise transformer modules [41]. Furthermore, BACFormer refines hybrid segmentation by introducing a boundary-aware hierarchical transformer topology that pairs explicit boundary perception with global context modeling to elevate overall delineation accuracy [20].

Existing hybrid CNN–Transformer architectures differ substantially in how convolutional and transformer features are integrated. While these approaches have improved segmentation performance, they adopt different strategies for combining local multi-scale representations and global contextual information, leaving opportunities for more effective architectural designs.

### Breast ultrasound image segmentation

Breast ultrasound imaging presents distinct challenges for automated segmentation due to severe speckle noise, acoustic shadows, and low contrast between target abnormalities and surrounding tissues. These imaging artifacts frequently produce poorly defined boundaries and highly variable lesion appearances, complicating precise delineation. The BUSI dataset introduced by Al-Dhabyani et al. has therefore become a widely adopted benchmark for breast ultrasound segmentation research [4]. Early investigations by Yap et al. highlighted the challenges of automated breast ultrasound lesion segmentation under heterogeneous imaging conditions [2]. Subsequently, studies by Alzahrani et al. [42] and more recent approaches have incorporated attention mechanisms, multi-scale feature extraction, boundary-aware refinement, and hybrid CNN–Transformer designs for breast ultrasound image segmentation [43–47]. However, effectively exploiting the complementary interaction between local multi-scale representations and global contextual information remains an active research challenge, motivating the design of MGT–UNet.

## Method

### Overview of MGT–UNet

**Fig. 1:**
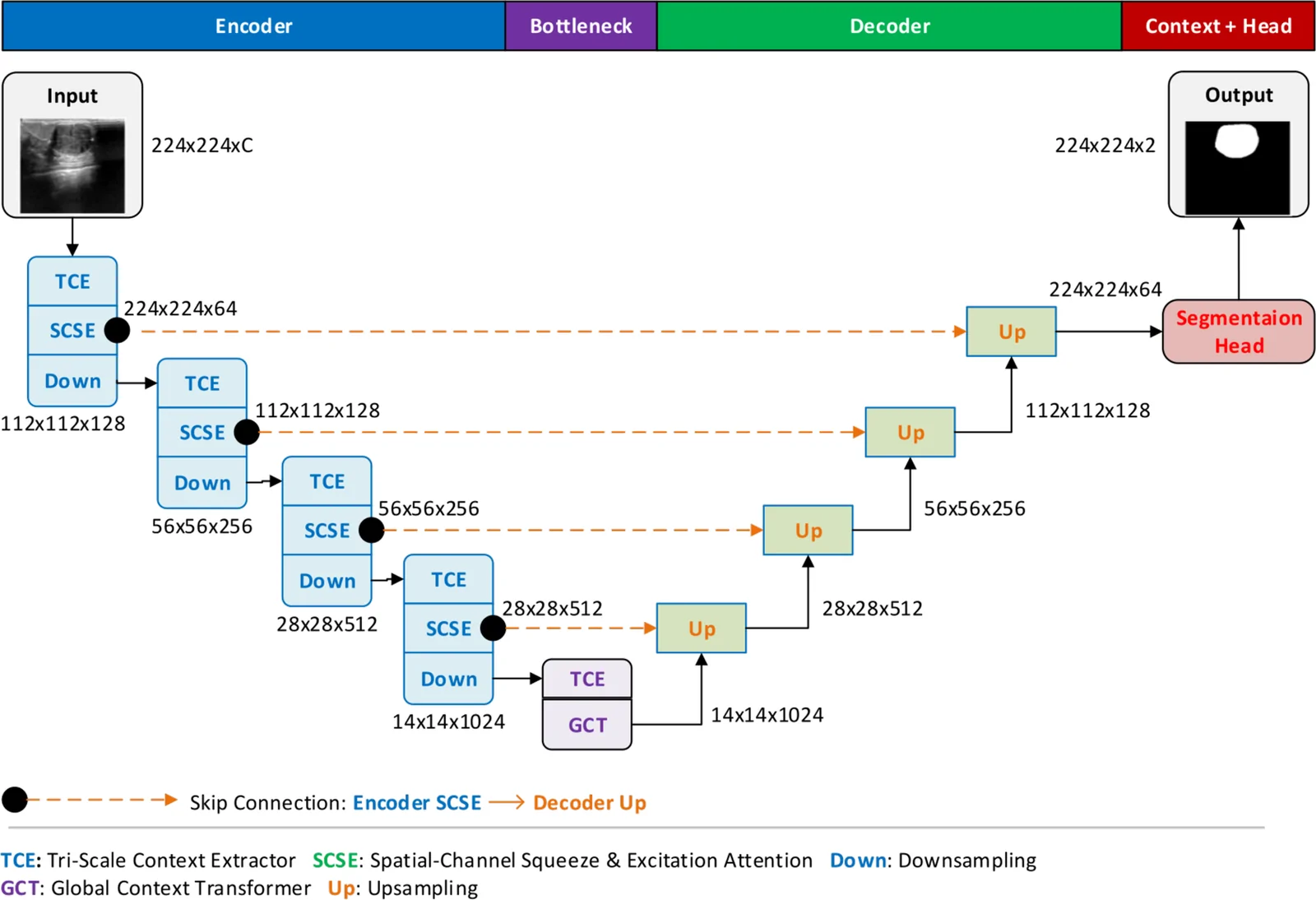
Overview of the proposed MGT–UNet architecture. The network follows a hierarchical encoder–decoder architecture with a transformer-based bottleneck. The encoder employs TCE blocks for hierarchical multi-scale feature extraction, while the bottleneck incorporates a GCT to model long-range contextual dependencies. Feature-map dimensions correspond to an example input size of *224×224* and are shown for illustration

The overall architecture of the proposed MGT–UNet is illustrated in Fig. 1. The network follows a hierarchical encoder–decoder structure that integrates multi-scale feature learning with global contextual modeling for breast ultrasound segmentation.

Given an input image $\mathbf{X}\in\mathbb{R}^{H\times W\times C}$, the encoder progressively extracts hierarchical feature representations through four encoder stages. Each stage consists of a TCE, followed by an SCSE attention module [26] and a downsampling block. The TCE learns complementary representations from multiple receptive fields, while SCSE recalibrates the extracted features using channel and spatial attention. The downsampling block consists of a *2×2* max-pooling operation followed by a double convolutional block (a sequence of two Conv–BN–ReLU blocks), where each convolutional block comprises convolution, batch normalization, and ReLU activation. This design progressively reduces the spatial resolution while increasing the feature dimension for hierarchical representation learning.

**Fig. 2:**
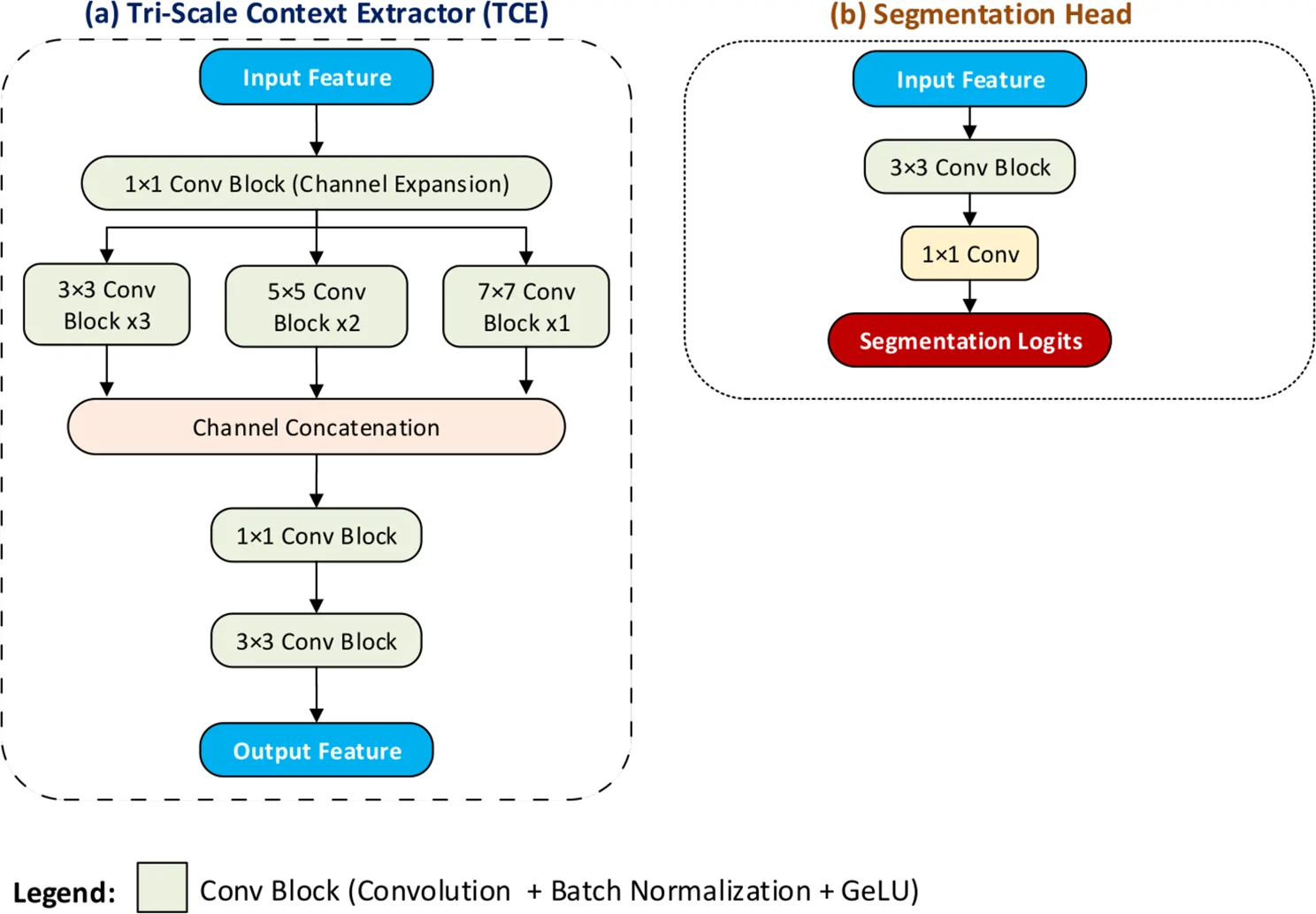
Key architectural components of MGT–UNet, including the TCE and the segmentation head

The deepest encoder feature is processed by the GCT, which models long-range contextual dependencies at low spatial resolution. During decoding, each stage first upsamples the feature map using a transposed convolution layer and then concatenates the corresponding encoder skip feature. A double convolutional block is subsequently applied to fuse the concatenated features and progressively reconstruct high-resolution representations. Finally, the reconstructed feature map is refined by a standard *3×3* convolutional block and projected to the desired number of segmentation classes through a *1×1* convolution. The network outputs segmentation logits directly without applying activation within the architecture (as shown in Fig. 2(b)).

Accordingly, the proposed MGT–UNet comprises two key components: the TCE, which extracts hierarchical multi-scale features through convolutional operations, and the GCT, which captures long-range contextual dependencies using transformer self-attention. Their integration enables the network to jointly exploit local spatial details and global semantic information during segmentation. Algorithm 1 summarizes the forward propagation procedure of MGT–UNet. The algorithm provides a high-level overview of the sequential operations performed during feature extraction, global contextual modeling, and decoder reconstruction before the detailed descriptions of the individual components presented in the following subsections. Here, $\mathrm{DoubleConv}(\cdot)$ denotes a sequence of two Conv–BN–ReLU blocks. Downsampling is performed using a *2×2* max-pooling operation, whereas each decoder block upsamples the feature maps using a transposed convolution, denoted by $\mathrm{ConvTranspose}(\cdot)$.

**Figure:**
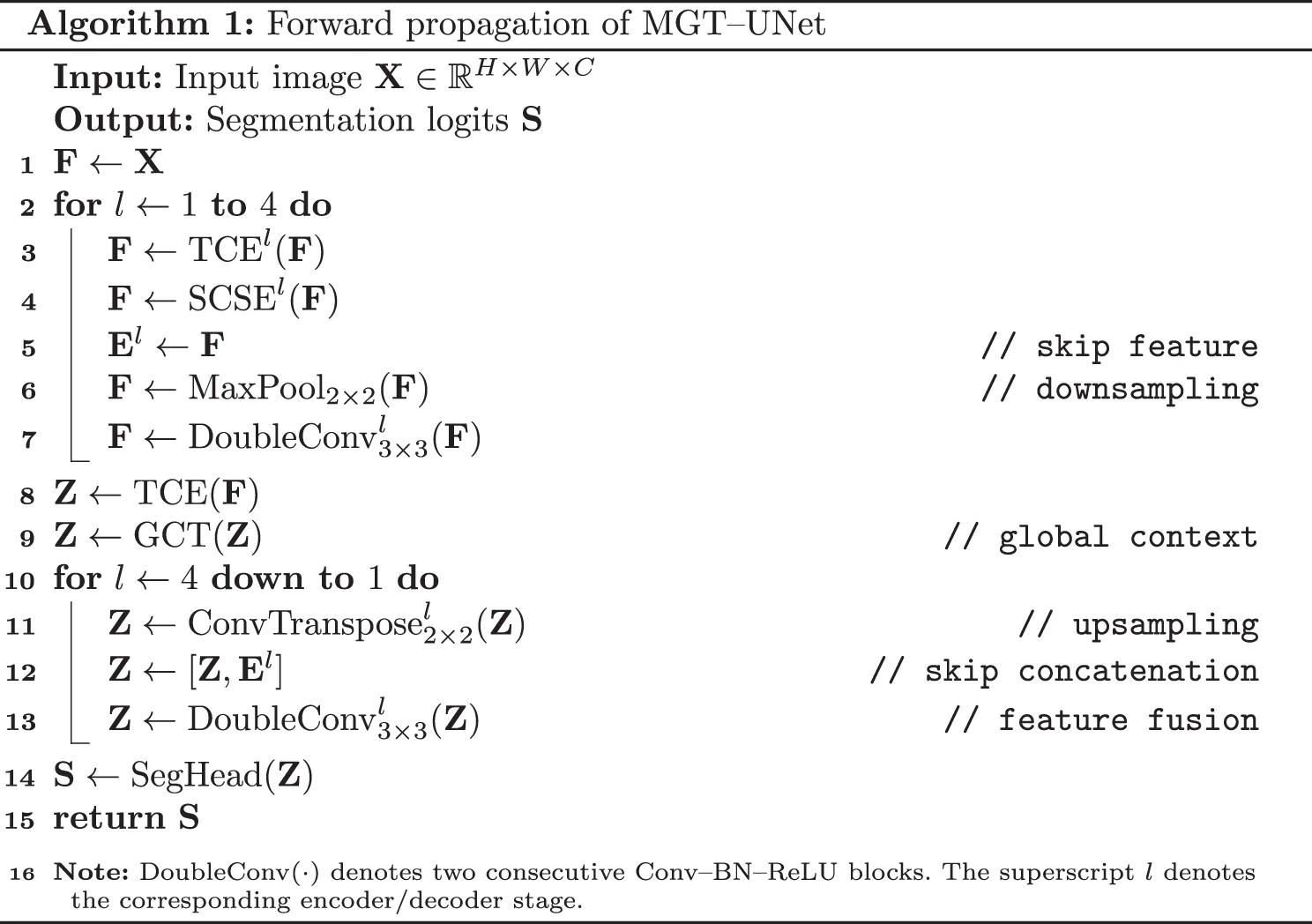

### Tri-scale context extractor

The structure of the TCE is illustrated in Fig. 2(a). TCE is designed to learn complementary spatial representations from three convolutional branches with different kernel sizes and depths. Given an input feature map $\mathbf{F}\in\mathbb{R}^{B\times C_{\mathrm{in}}\times H\times W}$, a *1×1* projection is first applied to map the input into a hidden feature space:

1 $$\mathbf{F}_{e}=\mathcal{C}_{1\times1}^{C_{\mathrm{in}}\rightarrow C_h}(\mathbf{F}),$$

where $\mathcal{C}_{k\times k}^{a\rightarrow b}(\cdot)$ denotes a Conv–BN–ReLU block with kernel size *k× k*, input channel dimension *a*, and output channel dimension *b*. The projected feature map is then processed by three parallel convolutional branches:

2 $$\begin{aligned}\mathbf{F}_{3}&=\mathcal{C}_{3\times3}^{C_h\rightarrow C_h}(\mathcal{C}_{3\times3}^{C_h\rightarrow C_h}(\mathcal{C}_{3\times3}^{C_h\rightarrow C_h}(\mathbf{F}_{e}))), \\\mathbf{F}_{5}&=\mathcal{C}_{5\times5}^{C_h\rightarrow C_h}(\mathcal{C}_{5\times5}^{C_h\rightarrow C_h}(\mathbf{F}_{e})), \\\mathbf{F}_{7}&=\mathcal{C}_{7\times7}^{C_h\rightarrow C_h}(\mathbf{F}_{e}).\end{aligned}$$

The three branch outputs are concatenated along the channel dimension:

3 $$\mathbf{F}_{c}=[\mathbf{F}_{3},\mathbf{F}_{5},\mathbf{F}_{7}]\in\mathbb{R}^{B\times 3C_h\times H\times W},$$

where *[·]* denotes channel-wise concatenation. The concatenated representation is then fused and refined using a *1×1* convolution followed by a *3×3* convolution:

4 $$\mathbf{F}_{r}=\mathcal{C}_{1\times1}^{3C_h\rightarrow C_{\mathrm{out}}}(\mathbf{F}_{c}),$$

5 $$\mathbf{F}_{\mathrm{out}}=\mathcal{C}_{3\times3}^{C_{\mathrm{out}}\rightarrow C_{\mathrm{out}}}(\mathbf{F}_{r}).$$

The *3×3* branch uses three consecutive convolutional blocks to capture fine local structures through progressive receptive-field expansion. The *5×5* branch uses two convolutional blocks to capture medium-scale spatial patterns, whereas the *7×7* branch captures broader contextual cues using a single larger-kernel convolutional block. By concatenating and fusing these branch outputs, TCE integrates complementary tri-scale representations into a unified feature map for subsequent encoder processing.

### Global context transformer

As illustrated in Fig. 1, the proposed GCT is positioned at the bottleneck stage of MGT–UNet. Unlike Vision Transformer (ViT)-based architectures that partition the input image into fixed-size patches before transformer processing, the proposed GCT operates directly on the bottleneck feature representation produced by the convolutional encoder. At this stage, the spatial resolution has already been substantially reduced while preserving rich semantic information, making global self-attention computationally efficient without requiring additional patch embedding or feature projection.

Let $\mathbf{F}_{b}\in\mathbb{R}^{B\times C\times H_b\times W_b}$ denote the bottleneck feature map, where $H_b=\frac{H}{16}$ and $W_b=\frac{W}{16}$ for the adopted architecture. Before global contextual modeling, the bottleneck feature is first refined by a TCE:

6 $$\mathbf{F}_{p}=\mathrm{TCE}_{b}(\mathbf{F}_{b}).$$

The refined bottleneck feature map is flattened into a sequence of spatial tokens:

7 $$\mathbf{X}=\operatorname{Flatten}(\mathbf{F}_{p})\in\mathbb{R}^{B\times N\times C},\qquad N=H_bW_b,$$

where *N* is the total number of spatial locations in the bottleneck feature map.

To preserve spatial information after flattening, a learnable two-dimensional positional embedding is employed. Separate row and column embeddings are learned and concatenated to construct the positional embedding for each spatial location:

8 $$\mathbf{P}=\operatorname{PosEmbed}(H_b,W_b)\in\mathbb{R}^{N\times C},$$

which is added to the token sequence before transformer encoding,

9 $$\mathbf{X}_0=\mathbf{X}+\mathbf{P}.$$

The position-aware token sequence is subsequently processed by a stack of *L* Transformer encoder layers:

10 $$\mathbf{X}_{L}=\operatorname{TransformerEncoder}(\mathbf{X}_{0}),$$

where $\operatorname{TransformerEncoder}(\cdot)$ denotes the standard Transformer encoder proposed by Vaswani et al. [11]. Finally, the refined token sequence is reshaped back to the spatial feature representation,

11 $$\mathbf{F}_{\mathrm{GCT}}=\operatorname{Reshape}(\mathbf{X}_{L})\in\mathbb{R}^{B\times C\times H_b\times W_b}.$$

Operating directly on the bottleneck feature map enables the GCT to model global contextual relationships across the entire image at the reduced bottleneck resolution. Since the convolutional encoder has already extracted hierarchical multi-scale features, the transformer focuses on learning long-range contextual dependencies without additional patch partitioning. Consequently, the proposed bottleneck design combines local convolutional representations with global contextual modeling for breast ultrasound segmentation.

### Training strategy

The proposed MGT–UNet is trained in a fully supervised manner using pixel-wise annotations. Given an input image $\mathbf{X}\in\mathbb{R}^{H\times W\times C}$ and its corresponding ground-truth mask $\mathbf{G}$, the network predicts segmentation logits $\mathbf{S}\in\mathbb{R}^{B\times K\times H\times W}$, where *B* denotes the batch size and *K=2* for the binary segmentation task considered in this study.

To jointly optimize pixel-wise classification accuracy and region-level overlap, the network is trained using a weighted combination of cross-entropy loss and Dice loss:

12 $$\mathcal{L}=\alpha\mathcal{L}_{\mathrm{CE}}+(1-\alpha)\mathcal{L}_{\mathrm{Dice}},$$

where $\alpha\in[0,1]$ controls the relative contributions of the two loss terms. In this study, *α=0.4* is adopted, assigning slightly greater weight to the Dice loss to emphasize region-overlap optimization, which is particularly important for accurate lesion segmentation in breast ultrasound images.

For the binary segmentation task, the network predicts logits for two classes. These logits are converted into class probabilities using the softmax function, where the predicted probability that pixel *i* belongs to class *k* is

13 $$P_{i,k}=\operatorname{Softmax}(\mathbf{s}_{i})_k,$$

where $\mathbf{s}_i$ denotes the predicted logit vector at pixel *i*, and $P_{i,k}$ is the corresponding probability assigned to class *k*.

The cross-entropy loss is computed as

14 $$\mathcal{L}_{\mathrm{CE}}=-\frac{1}{N}\sum_{i=1}^{N}\sum_{k=1}^{K}G_{i,k}\log(P_{i,k}),$$

where *N* is the total number of pixels, *K* is the number of classes, $P_{i,k}$ denotes the predicted probability of class *k* at pixel *i*, and $G_{i,k}\in\{0,1\}$ is the corresponding one-hot encoded ground-truth label.

The Dice loss is defined as

15 $$\mathcal{L}_{\mathrm{Dice}}=1-\frac{1}{K}\sum_{k=1}^{K}\frac{2\sum_i P_{i,k}G_{i,k}+\epsilon}{\sum_i P_{i,k}+\sum_i G_{i,k}+\epsilon},$$

where *ε* is a small positive constant introduced for numerical stability.

## Experiments

### Experimental setup

All models were implemented using the PyTorch deep learning framework and trained under a unified hardware and software environment. To ensure fair comparison and reproducibility, all methods shared identical software dependencies, preprocessing pipelines, optimization strategies, and evaluation protocols. Detailed implementation information, including the hardware configuration and software libraries, is provided in Appendix A.

The same MGT–UNet architecture, loss function, training strategy, and hyperparameter configuration described in Sects. 3.4 and 4.5 were consistently applied to the BUSI, ISIC2018, and Synapse datasets. No dataset-specific architectural modifications or hyperparameter tuning were performed. To minimize the impact of training randomness, independent experiments were conducted for each dataset using different random seeds. Results are reported as the mean *±* standard deviation to provide a more reliable performance estimate.

Segmentation performance was evaluated using the Dice coefficient (Dice), Intersection-over-Union (IoU), and the 95th percentile Hausdorff Distance (HD$_{95}$). Dice and IoU quantify the overlap between the predicted segmentation and the ground truth, whereas HD$_{95}$ measures boundary discrepancy while reducing the influence of extreme outliers. Together, these complementary metrics provide a comprehensive assessment of both region-level segmentation accuracy and boundary delineation quality.

### Datasets

The proposed MGT–UNet is evaluated on three publicly available medical image segmentation datasets representing ultrasound, dermoscopy, and CT imaging modalities. The BUSI dataset [4] serves as the primary evaluation benchmark because breast ultrasound segmentation is the target application of this study. The ISIC2018 dermoscopic lesion dataset [5, 6] and the Synapse dataset [7] are included as evaluations to assess the proposed architecture on different imaging modalities under a consistent binary segmentation protocol. The dataset partitions are generated once, kept fixed throughout all experiments, and shared across all compared methods. For multi-seed evaluations, only the random model initialization and stochastic optimization vary. A summary of the datasets, data splits, and segmentation tasks is presented in Table 1.

**Table 1:** Summary of the datasets, data splits, and segmentation tasks used in this study

Dataset	Modality	Train	Val	Test	Segmentation Task
BUSI	Ultrasound	546	117	117	Breast lesion
ISIC2018	Dermoscopy	2594	100	1000	Skin lesion
Synapse	CT	706	151	152	Abdominal structure

### Evaluation metrics

The Dice is adopted as the primary evaluation metric, while the IoU, HD$_{95}$, Precision, and Recall provide complementary assessments of region overlap, boundary accuracy, and pixel-wise classification performance. Since all experiments adopt a binary segmentation protocol, all metrics are computed for the positive target class in each dataset.

Let *TP*, *FP*, *TN*, and *FN* denote the numbers of true-positive, false-positive, true-negative, and false-negative pixels, respectively. Region-overlap metrics are then defined as

16 $$\mathrm{Dice}=\frac{2TP}{2TP+FP+FN},$$

17 $$\mathrm{IoU}=\frac{TP}{TP+FP+FN}.$$

Pixel-wise Precision and Recall are computed as

18 $$\mathrm{Precision}=\frac{TP}{TP+FP},\qquad\mathrm{Recall}=\frac{TP}{TP+FN}.$$

Boundary accuracy is assessed using the 95th-percentile Hausdorff distance (HD$_{95}$):

19 $$\mathrm{HD}_{95}=\operatorname{percentile}_{95}\left(\mathcal{D}(\mathcal{X},\mathcal{Y})\cup\mathcal{D}(\mathcal{Y},\mathcal{X})\right),$$

where $\mathcal{X}$ and $\mathcal{Y}$ denote the boundary point sets of the predicted and ground-truth masks, respectively, and $\mathcal{D}(\cdot,\cdot)$ denotes the Euclidean surface distance.

To ensure reproducible evaluation, Algorithm 2 summarizes the metric computation procedure adopted in this study. Metrics are computed independently for each test image, with explicit handling of cases in which the ground-truth or predicted mask contains no positive target, as commonly encountered in the BUSI dataset due to the inclusion of normal breast ultrasound images. Dice, IoU, Precision, and Recall are averaged over all test images, whereas HD$_{95}$ is averaged only over valid cases in which both the predicted and ground-truth masks contain positive target regions. This protocol ensures consistent treatment of normal images while providing a reliable assessment of both region overlap and boundary accuracy.

**Figure:**
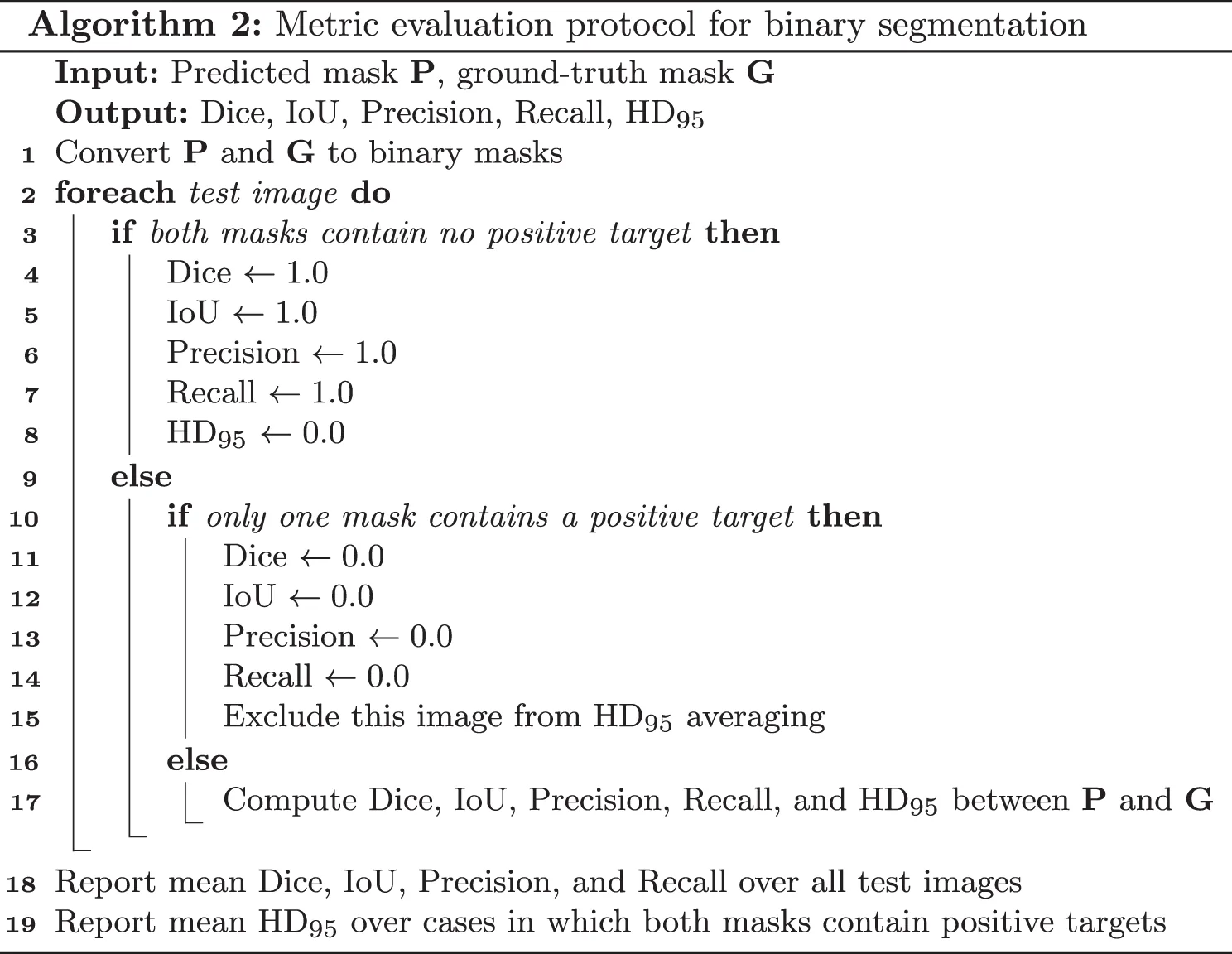

### Implementation and training protocol

All input images were resized to a fixed spatial resolution of *224×224* prior to training and inference. Adopting a unified resolution ensures consistent computational complexity across all evaluated methods, enabling a fair comparison of model capacity, memory consumption, and execution time. To guarantee reproducibility, identical training, validation, and test partitions were maintained for all experiments. Furthermore, each model was trained independently across multiple random initializations to account for stochastic variations arising from weight initialization, data shuffling, and optimization. Performance metrics are reported as the mean *±* standard deviation across all runs.

All baseline models were implemented strictly according to their original publications. Wherever available, official source codes were adopted; otherwise, the architectures were meticulously re-implemented following the exact specifications in the corresponding papers. To ensure a controlled evaluation, all methods shared an identical preprocessing pipeline, optimization strategy, hyperparameter configuration, and evaluation protocol. Consequently, the network architecture remains the sole variable driving performance differences among the compared methods. Notably, no data augmentation techniques were employed, isolating the influence of the architectural design from the confounding effects of regularization strategies. The loss function is described in Sect. 3.4, while the optimization procedure and hyperparameter selection protocol are presented in Sect. 4.5.

### Hyperparameter selection

To determine the optimization and architectural hyperparameters of MGT–UNet, sensitivity analyses were conducted exclusively on the BUSI validation set. Each hyperparameter was varied independently while all remaining settings were kept fixed. The reported metrics in Tables 2, 3, and 4 are validation results and were used solely for hyperparameter selection. The test set was not used during this process and was reserved exclusively for the final performance evaluation reported in Sect. 5.

**Table 2:** Sensitivity analysis of the initial learning rate on the BUSI validation set used for hyperparameter selection

LR$_{\mathrm{base}}$	Dice$_{\mathrm{val}}$ (%) *↑*	IoU$_{\mathrm{val}}$ (%) *↑*	HD$_{95,\mathrm{val}}$*↓*	Epoch$_{\mathrm{best}}$*↓*
$1\times10^{-3}$	79.68	71.97	20.10	47
$5\times10^{-3}$	80.84	73.88	18.27	42
$1\times10^{-2}$	80.70	73.63	16.27	33

Note: LR_base_: initial learning rate. Dice_val_: validation Dice. IoU_val_: validation IoU.HD_95,val_: validation HD_95_. Epoch_best:_ epoch corresponding to the highest validationDice. ↑ and ↓ indicate that higher and lower values are preferred, respectively

**Table 3:** Sensitivity analysis of the base feature dimension on the BUSI validation set used for hyperparameter selection

C$_{\mathrm{base}}$	C$_{\mathrm{encoder}}$	Dice$_{\mathrm{val}}$ (%) *↑*	IoU$_{\mathrm{val}}$ (%) *↑*	HD$_{95,\mathrm{val}}$*↓*	Params (M) *↓*
32	32|64|128|256|512	78.75	71.37	18.22	59.50
64	64|128|256|512|1024	80.84	73.88	18.27	237.89

Note: C_base_: base number of feature channels. C_encoder_: channel dimensions of the encoder stages. Dice_val_: validation Dice. IoU_val_: validation IoU. HD_95,val_: validation HD_95_. Params (M): number of trainable parameters in millions. ↑ and ↓ indicate that higher and lower values are preferred, respectively

**Table 4:** Senvalidation results and were used solelysitivity analysis of the transformer configuration on the BUSI validation set used for hyperparameter selection. Only the transformer depth and the number of attention heads are varied, while all other settings are kept fixed

Transformer Configuration	Dice$_{\mathrm{val}}$ (%) *↑*	IoU$_{\mathrm{val}}$ (%) *↑*	HD$_{95,\mathrm{val}}$*↓*	Params (M) *↓*
Depth = 1, Heads = 2	80.84	73.88	18.27	237.89
Depth = 2, Heads = 4	80.38	73.22	18.24	250.48

Note: Depth: number of Transformer encoder layers. Heads: number of multi-head self-attention heads. Dice_val_: validation Dice. IoU_val_: validation IoU. HD_95,val_: validation HD_95_. Params (M): number of trainable parameters in millions. ↑ and ↓ indicate that higher and lower values are preferred, respectively

The final model is trained using stochastic gradient descent (SGD) with a momentum of 0.9 and a weight decay of $10^{-4}$. The learning rate follows a polynomial decay schedule,

$$\eta_t=\eta_0\left(1-\frac{t}{T}\right)^{0.9},$$

where $\eta_0$ is the initial learning rate and *t* and *T* denote the current and total training iterations, respectively. The final hyperparameter configuration selected from the validation experiments is summarized in Table 5.

**Table 5:** Final hyperparameter configuration of MGT–UNet selected based on the BUSI validation set

Category	Parameter	Value
Optimizer	SGD	-
Learning rate	Initial LR	$5\times10^{-3}$
Momentum		0.9
Weight decay		$10^{-4}$
Training	Maximum epochs	300
Training	Early stopping	Patience = 50 epochs$^{a}$
Scheduler	Polynomial	power = 0.9
Input size		*224×224*
Base channels		64
Transformer layers		1
Attention heads		2
Dropout		0.1

^a^Training is terminated if the validation Dice does not improve for 50consecutive epochs. The model achieving the highest validation Dice isretained for testing.

### Statistical analysis

The statistical analysis in this study serves two complementary purposes: quantifying training reproducibility and evaluating whether the proposed MGT-UNet achieves statistically significant improvements over the strongest baseline. To characterize the variability introduced by stochastic optimization, each model is trained repeatedly using different random seeds while maintaining identical dataset partitions and training hyperparameters. Segmentation performance is therefore reported as the mean *±* standard deviation across these independent training runs. These repeated runs are used solely to estimate optimization reproducibility and are not regarded as independent observations for statistical hypothesis testing.

Statistical significance is evaluated using paired image-level Dice coefficients on the BUSI test set. Because Dice is intended to quantify segmentation quality for images containing lesions, the paired statistical analysis is performed only on lesion-present test images. Images with empty ground-truth masks are excluded from hypothesis testing to avoid conflating lesion segmentation performance with false-positive suppression on normal images.

Let *N* denote the number of lesion-present test images and *K* the number of repeated training runs. For each training run, MGT-UNet and each baseline model are trained independently using identical experimental settings except for the random seed. Each trained model produces one Dice coefficient for every test image, yielding *K* Dice measurements for each image.

To avoid pseudoreplication, repeated training runs are not treated as independent observations. Instead, for each test image *i*, the Dice coefficients obtained from the *K* training runs are averaged,

20 $$\bar{D}_i=\frac{1}{K}\sum_{k=1}^{K}D_{ik},$$

where $D_{ik}$ denotes the Dice coefficient of image *i* obtained from the model trained with random seed *k*. The same averaging procedure is applied to both MGT-UNet and the strongest baseline (UNet++), resulting in one paired observation for each test image,

21 $$\left(\bar{D}^{\mathrm{MGT}}_i,\bar{D}^{\mathrm{UNet++}}_i\right),\qquad i=1,\ldots,N.$$

Accordingly, the effective sample size equals the number of lesion-present test images rather than the number of repeated training runs. The paired Dice difference for each image is computed as

22 $$\Delta_i=\bar{D}^{\mathrm{MGT}}_i-\bar{D}^{\mathrm{UNet++}}_i,\qquad i=1,\ldots,N.$$

The average paired Dice difference is reported together with its 95% confidence interval (CI),

23 $$\bar{\Delta}=\frac{1}{N}\sum_{i=1}^{N}\Delta_i,$$

where the confidence interval is estimated using the paired-sample *t* distribution. The corresponding paired effect size is quantified by Cohen’s $d_z$.

Before hypothesis testing, the normality of the paired Dice differences is evaluated using the Shapiro–Wilk test [48]. If the normality assumption is satisfied, a paired *t*-test is performed [49]; otherwise, the non-parametric Wilcoxon signed-rank test is employed [50]. A significance level of *p < 0.05* is adopted for all statistical tests [49, 51].

### Computational efficiency

Computational efficiency is evaluated to complement the segmentation performance analysis. The reported metrics include the number of trainable parameters (Params), floating-point operations (GFLOPs), average inference time per image, frames per second (FPS), peak GPU memory consumption during inference, the epoch achieving the best validation performance, and the corresponding training time.

All models are evaluated using the same hardware platform, software environment, input resolution (*224×224*), and a batch size of one. Model parameters are obtained directly from the network architecture, and GFLOPs are computed using an identical profiling configuration for all models. Average inference time is calculated by dividing the total forward-pass time over the BUSI test set by the number of test images, while FPS is computed as its reciprocal. Peak GPU memory consumption is recorded during inference, and training time is measured from the start of training until the checkpoint with the highest validation Dice is obtained. Using an identical evaluation protocol ensures that the reported computational metrics primarily reflect differences in network architecture rather than implementation or preprocessing.

## Results

### Quantitative performance comparison

Table 6 compares MGT–UNet with representative CNN-, Transformer-, and hybrid segmentation models across the three evaluation datasets. Detailed quantitative results on the primary BUSI benchmark are presented in Table 7.

**Table 6:** Quantitative comparison with representative and recent medical image segmentation methods. Results are reported as mean *±* standard deviation (%)

	Dice *↑*		
Model	BUSI	ISIC2018	Synapse
U-Net(2015)	72.59 *±* 2.90	85.53 *±* 0.41	75.60 *±* 1.17
DeepLabV3+(2018)	69.35 *±* 2.57	85.77 *±* 0.57	68.91 *±* 0.82
UNet++(2020)	74.02 *±* 1.41	85.88 *±* 0.32	76.11 *±* 0.57
TransUNet(2021)	68.18 *±* 4.05	87.67 *±* 0.53	76.07 *±* 1.53
Swin-Unet(2023)	47.02 *±* 5.45	85.94 *±* 0.92	57.66 *±* 0.50
BACFormer(2026)	69.07 *±* 3.02	86.88 *±* 0.44	69.58 *±* 1.59
MGT–UNet (Ours)	77.74 *±* 1.41	86.98 *±* 0.34	71.68 *±* 0.78

**Table 7:** Detailed quantitative comparison on the BUSI breast ultrasound dataset. Results are reported as mean *±* standard deviation. *↑* and *↓* indicate that higher and lower values are preferred, respectively

Model	Dice (%)*↑*	IoU (%)*↑*	HD$_{95}$*↓*	Precision (%)*↑*	Recall (%)*↑*
U-Net(2015)	72.59 *±* 2.90	65.91 *±* 3.23	23.90 *±* 2.83	74.56 *±* 4.42	75.21 *±* 2.48
DeepLabV3+(2018)	69.35 *±* 2.57	62.68 *±* 2.76	23.87 *±* 3.59	71.40 *±* 3.17	71.84 *±* 1.58
UNet++(2020)	74.02 *±* 1.41	68.12 *±* 1.50	20.63 *±* 2.58	76.48 *±* 2.08	75.65 *±* 1.55
TransUNet(2021)	68.18 *±* 4.05	61.65 *±* 4.27	19.86 *±* 4.73	69.22 *±* 4.88	70.90 *±* 2.94
Swin-Unet(2023)	47.02 *±* 5.45	38.33 *±* 5.59	48.41 *±* 6.88	47.84 *±* 6.91	57.70 *±* 3.26
BACFormer(2026)	69.07 *±* 3.02	62.47 *±* 3.44	22.89 *±* 2.31	69.48 *±* 4.41	73.60 *±* 1.40
MGT–UNet (Ours)	77.74 *±* 1.41	71.24 *±* 1.39	18.11 *±* 1.36	80.35 *±* 1.58	79.16 *±* 1.50

On the BUSI breast ultrasound dataset, MGT–UNet achieves the best overall performance, obtaining a Dice of 77.74%, an IoU of 71.24%, and an HD$_{95}$ of 18.11. Compared with the strongest baseline, UNet++, the proposed model improves the Dice by 3.72 percentage points while also achieving the lowest HD$_{95}$. Together with the highest Precision and Recall among all evaluated methods, these results indicate better overlap- and boundary-based performance than the compared approaches. Compared with the recent hybrid CNN–Transformer architecture BACFormer, MGT–UNet also achieves higher mean performance on the BUSI dataset. These results suggest that combining multi-scale local feature extraction with bottleneck-level global contextual modeling is beneficial for breast ultrasound image segmentation.

On the ISIC2018 dataset, MGT–UNet achieves a Dice of 86.98%, which is lower than TransUNet but higher than several other evaluated methods. Although it does not achieve the highest Dice, the results indicate that the proposed architecture remains effective under dermoscopic imaging conditions. The evaluation on the Synapse dataset further examines the behavior of MGT–UNet under a different imaging modality using the same binary segmentation protocol. While the proposed model does not outperform the strongest baseline on this dataset, the results provide complementary evidence of its performance under a unified binary segmentation setting and should not be interpreted as results on the standard multi-class Synapse benchmark.

### Training dynamics

**Fig. 3:**
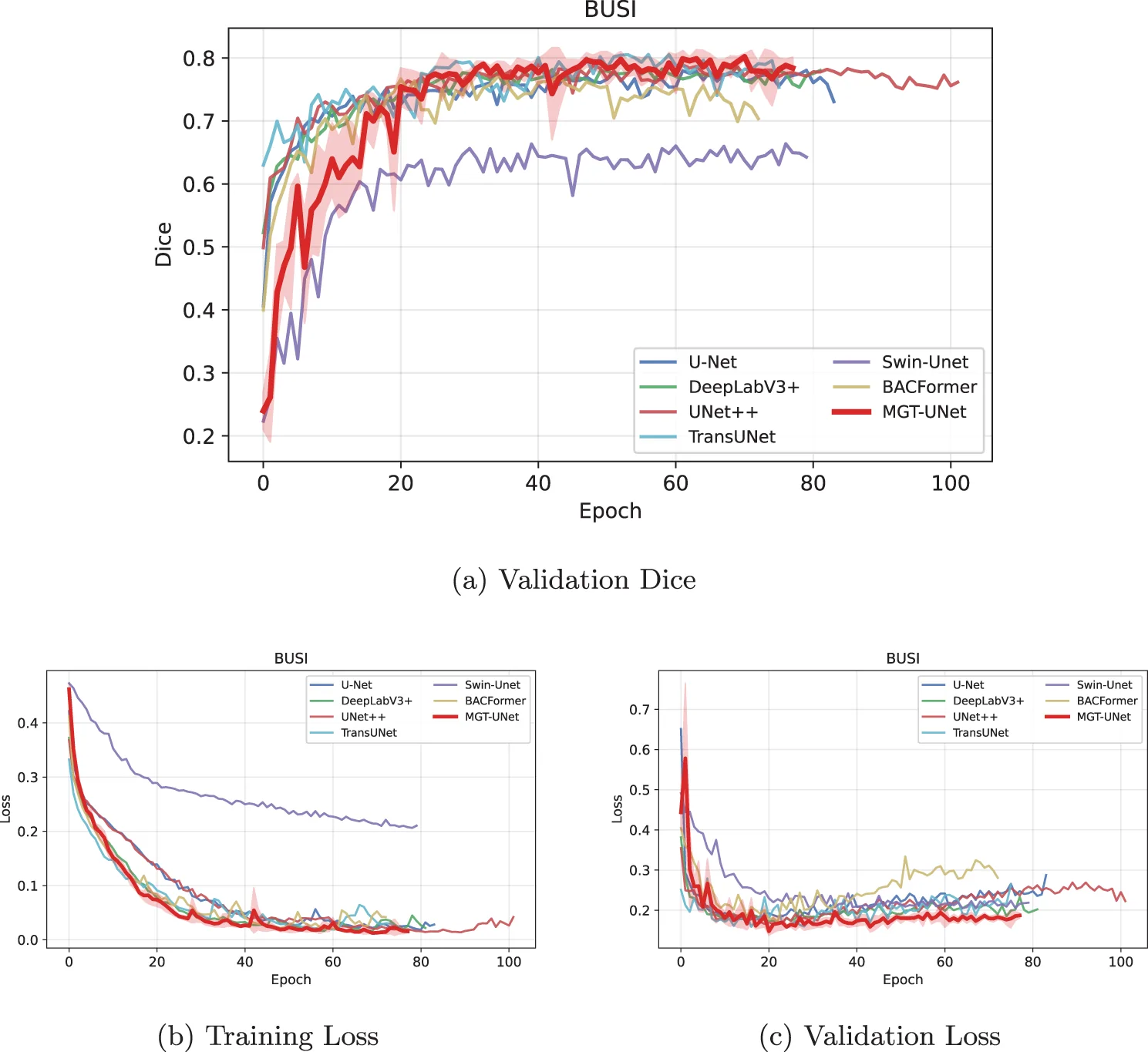
Training dynamics on the BUSI dataset. Solid curves show mean values, and shaded regions indicate standard deviation

**Fig. 4:**
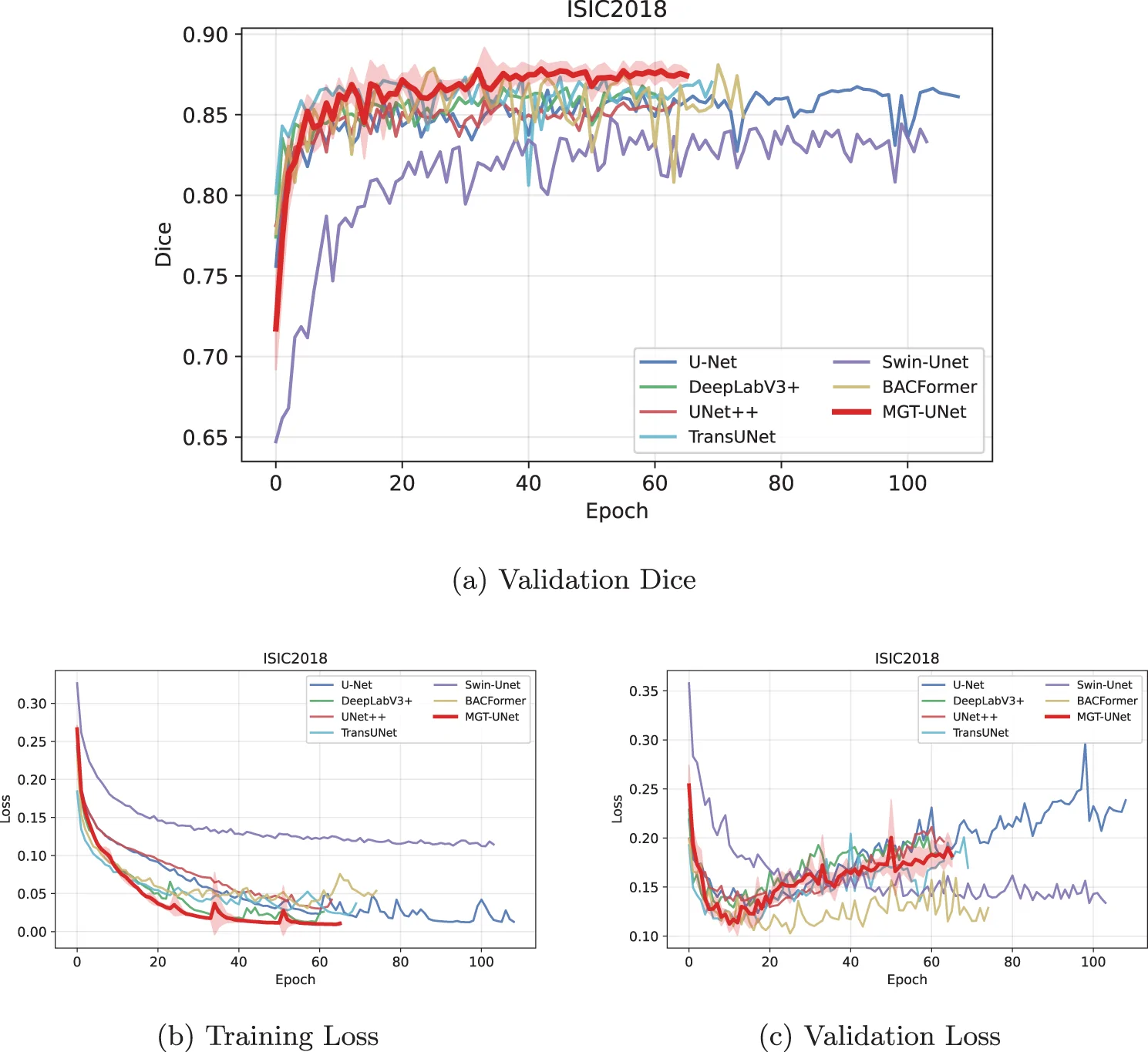
Training dynamics on the ISIC2018 dataset. Solid curves show mean values, and shaded regions indicate standard deviation

**Fig. 5:**
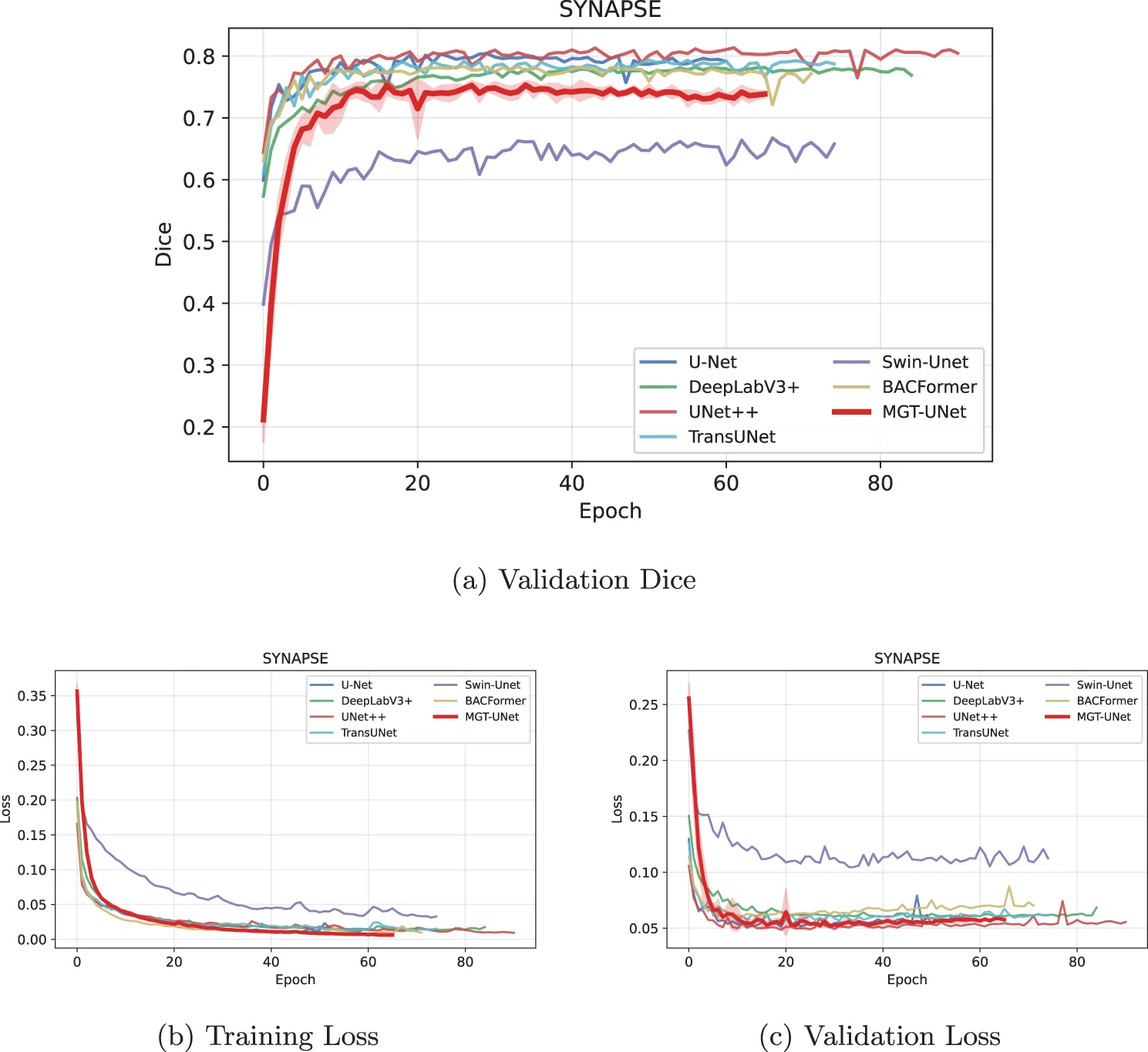
Training dynamics on the Synapse dataset. Solid curves show mean values, and shaded regions indicate standard deviation

To further analyze the optimization behavior of the proposed model, Figs. 3, 4, and 5 illustrate the training dynamics on the BUSI, ISIC2018, and Synapse datasets. Each curve represents the mean performance across independent training runs, while the shaded region indicates the corresponding standard deviation. Although the maximum training length was set to 300 epochs, early stopping (Table 5) terminated training after the validation Dice failed to improve for the specified patience period. Consequently, the training curves may have different lengths.

On the BUSI dataset, MGT–UNet exhibits stable optimization during training. Although its validation Dice increases more gradually during the initial training stage than several baseline methods, the performance improves steadily and eventually reaches the highest validation Dice among the evaluated methods (Fig. 3a). The model reaches its best validation performance after approximately 53 epochs on average, which is consistent with the convergence statistics reported in Table 9. The corresponding training and validation loss curves decrease smoothly with limited oscillation, indicating stable optimization with no obvious signs of severe overfitting before early stopping. These observations are consistent with the quantitative results in Table 7, where MGT–UNet achieves the highest segmentation accuracy on the BUSI dataset.

On the ISIC2018 and Synapse datasets, MGT–UNet also exhibits stable optimization throughout training. On ISIC2018, most models converge rapidly in terms of validation Dice, with relatively small variation after the early training stage, and MGT–UNet follows a similarly stable convergence trajectory. Since the validation loss curves display atypical behavior for several models on ISIC2018, the interpretation of optimization behavior is based primarily on the validation Dice curves. On the Synapse dataset, both the training and validation losses decrease smoothly during optimization. Although the final validation Dice is lower than that of the strongest baseline methods, this observation is consistent with the quantitative results presented in Sect. 5.1.

### Statistical significance

Following the statistical analysis protocol described in Sect. 4.6, image-level paired analysis was performed on lesion-present BUSI test images. Images with empty ground-truth masks were excluded to ensure that the Dice reflected lesion segmentation performance rather than false-positive suppression on normal images.

As summarized in Table 8, MGT–UNet achieved a higher mean Dice than UNet++, corresponding to an average paired improvement of 3.46 percentage points. The estimated 95% confidence interval of the mean paired Dice difference ranged from 0.40 to 6.51 percentage points, while the paired effect size was small-to-moderate ($d_z=0.236$).

**Table 8:** Image-level paired statistical analysis on lesion-present BUSI test images. Mean diff. denotes the mean paired Dice difference between MGT–UNet and UNet++. CI denotes the 95% confidence interval. The statistical test was selected according to the procedure described in Sect. 4.6

Comparison	*N*	Diff.	95% CI	Test	*p*	$d_z$
MGT–UNet vs. UNet++	91	3.46	[0.40, 6.51]	Wilcoxon	0.690	0.236

The Shapiro–Wilk test indicated that the paired Dice differences deviated significantly from a normal distribution (*p < 0.001*). Therefore, according to the predefined statistical protocol, the Wilcoxon signed-rank test was selected for hypothesis testing. The resulting *p*-value (*p=0.690*) indicates that the observed improvement was not statistically significant at the 5% significance level.

**Fig. 6:**
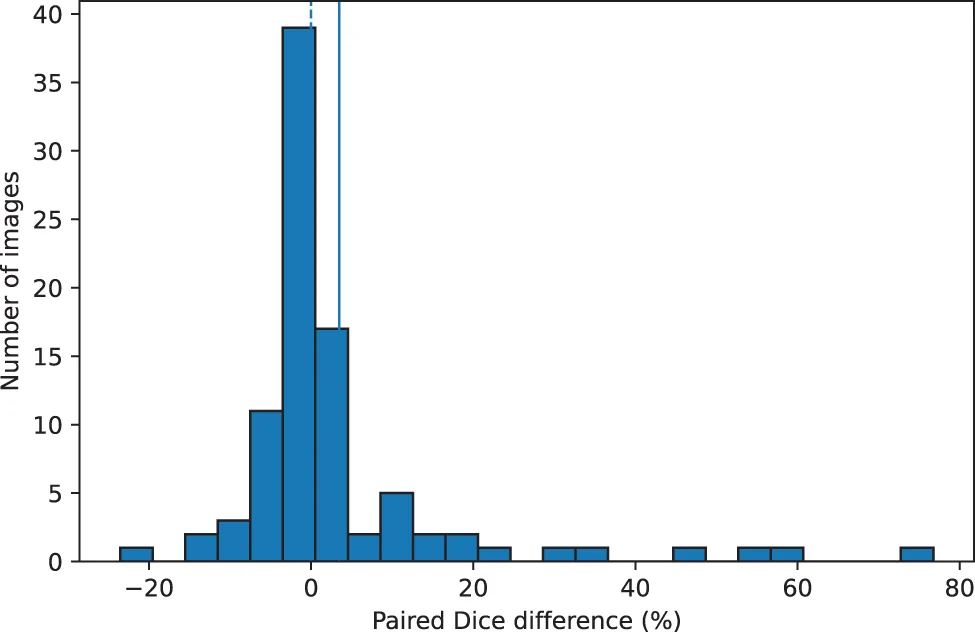
Distribution of paired Dice differences between MGT–UNet and UNet++ on lesion-present BUSI test images. Positive values indicate images for which MGT–UNet outperforms UNet++

To better understand this result, the paired image-level Dice differences were further examined. MGT–UNet achieved higher averaged Dice coefficients than UNet++ on 44 lesion-present images, lower Dice scores on another 44 images, and identical averaged Dice coefficients on the remaining 3 images. Consequently, the improvement in the average Dice was primarily driven by relatively large gains on a subset of challenging cases rather than by consistent improvements across the entire test set. Figure 6 illustrates the distribution of paired Dice differences between MGT–UNet and UNet++. Most differences are concentrated near zero, whereas a relatively small number of images exhibit substantial positive improvements. Representative paired-case visualizations are provided in Appendix B.

### Performance stability

**Fig. 7:**
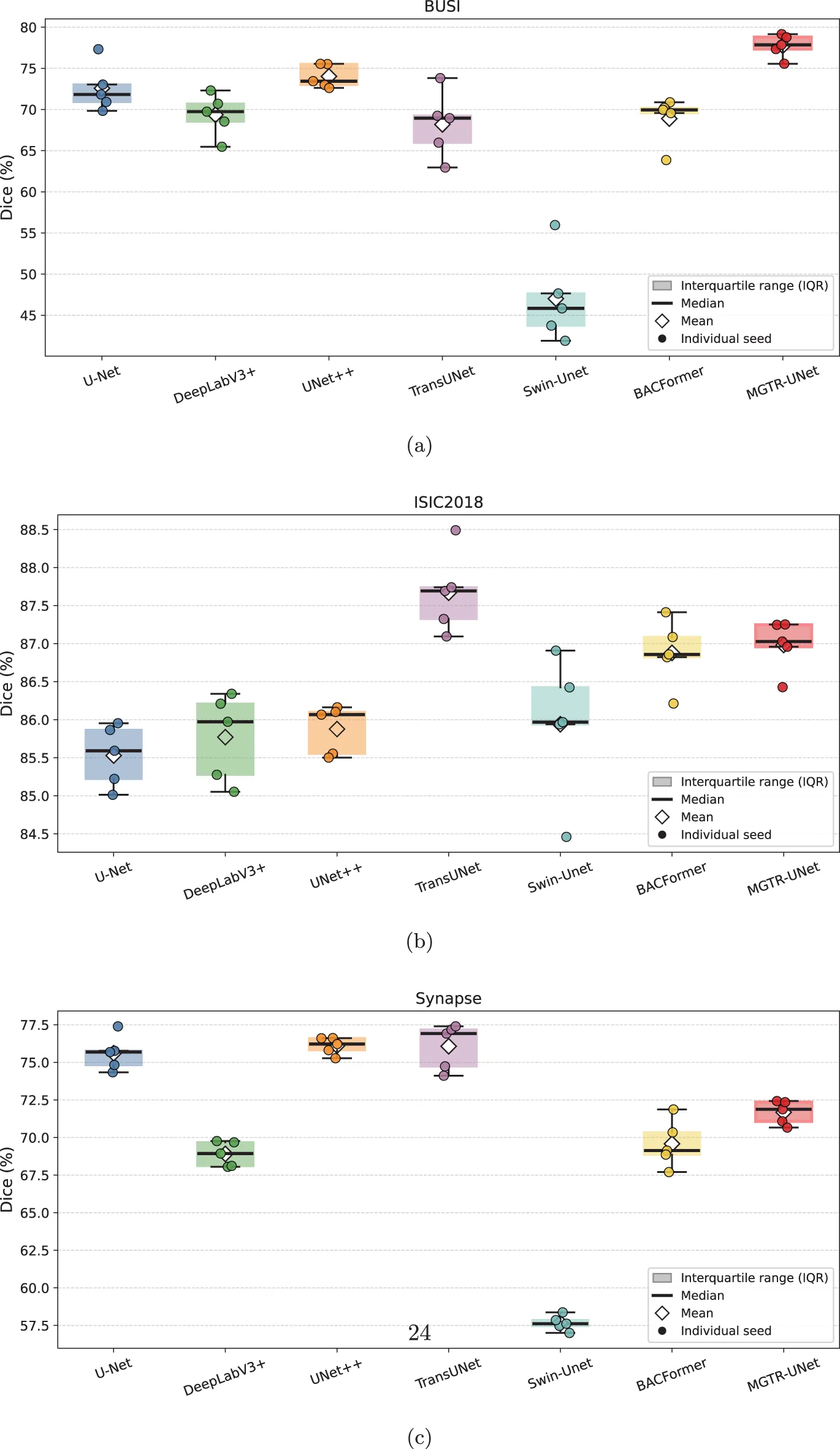
Distribution of test Dice coefficients across repeated training runs. Boxes represent the interquartile range (IQR), horizontal lines indicate the median, diamonds denote the mean, and individual points correspond to independent training runs with different random initializations

Figure 7 presents the distribution of the final test Dice coefficients obtained from repeated training runs. These distributions provide an assessment of the reproducibility of the final segmentation performance under different random initializations and complement the image-level statistical significance analysis.

On the BUSI dataset (Fig. 7a), MGT–UNet achieves the highest median Dice while exhibiting a relatively compact interquartile range. Although small variations are observed across different random initializations, the performance distribution remains consistently above those of the compared methods, indicating that the reported improvement is reproducible across repeated training runs.

On the ISIC2018 and Synapse datasets (Fig. 7b and c), the performance distributions also remain relatively compact across repeated runs. While MGT–UNet does not achieve the highest median Dice on these datasets, the limited variation indicates that the final segmentation performance is reproducible under different random initializations.

### Qualitative analysis

**Fig. 8:**
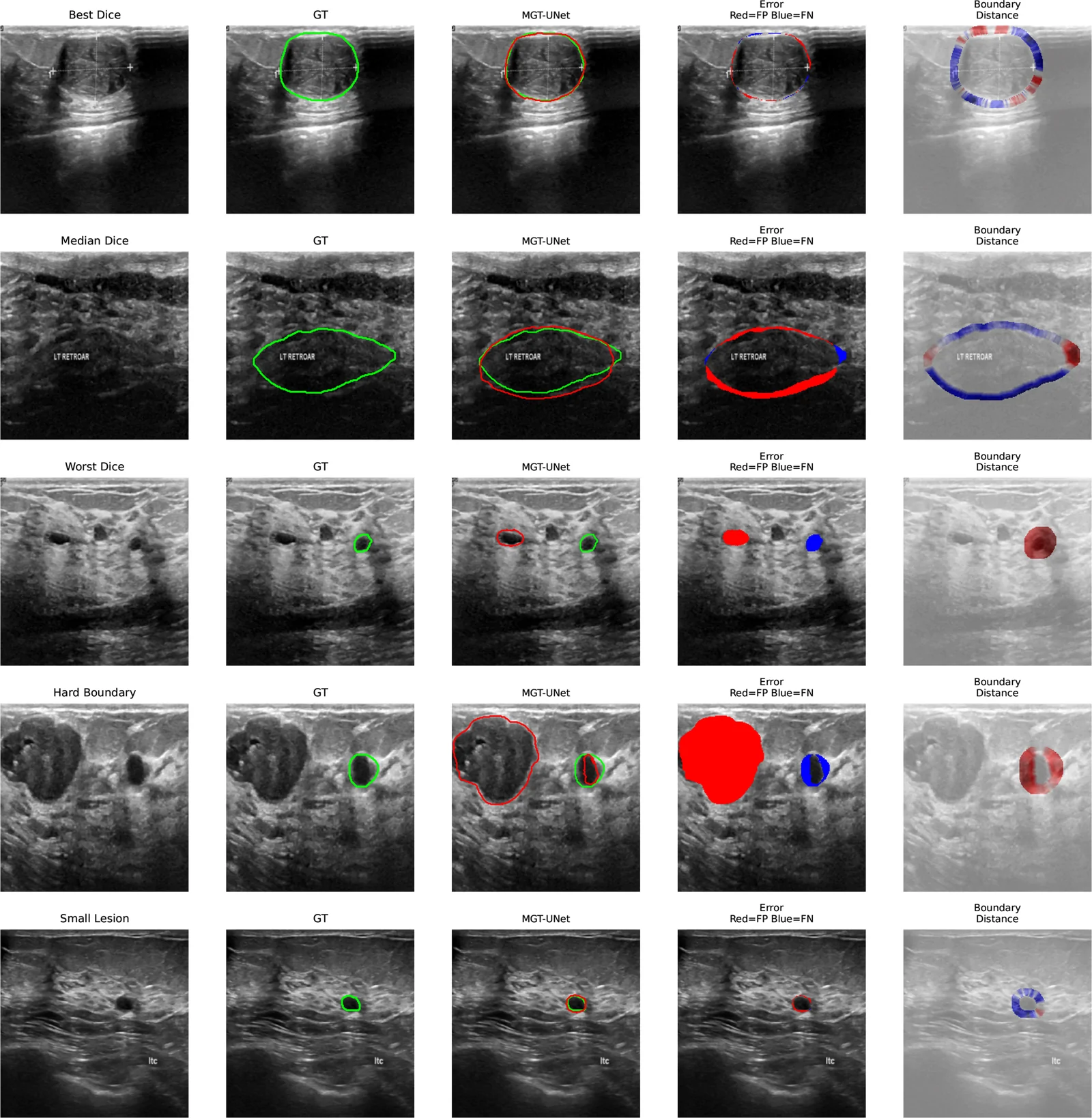
Qualitative segmentation results on the BUSI dataset. Representative cases include the best, median, and worst Dice coefficients together with hard-boundary and small positive target examples. Columns show the input image, ground-truth annotation (GT), MGT–UNet prediction, error map (red: false positive; blue: false negative), and signed boundary-distance visualization

**Fig. 9:**
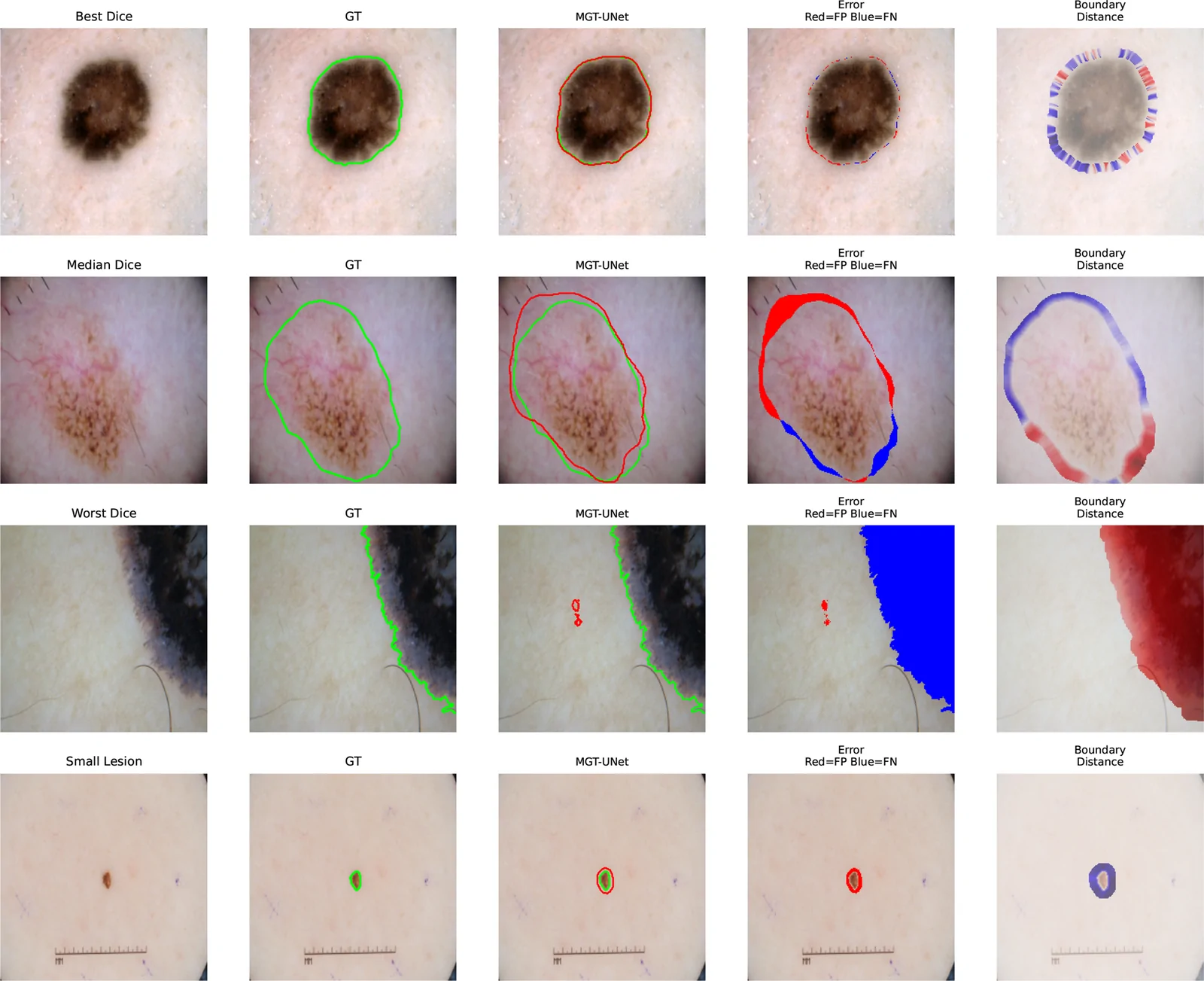
Qualitative segmentation results on the ISIC2018 dataset. Representative cases include the best, median, and worst Dice coefficients together with a small-lesion example. Columns show the input image, GT, MGT–UNet prediction, error map, and signed boundary-distance visualization

**Fig. 10:**
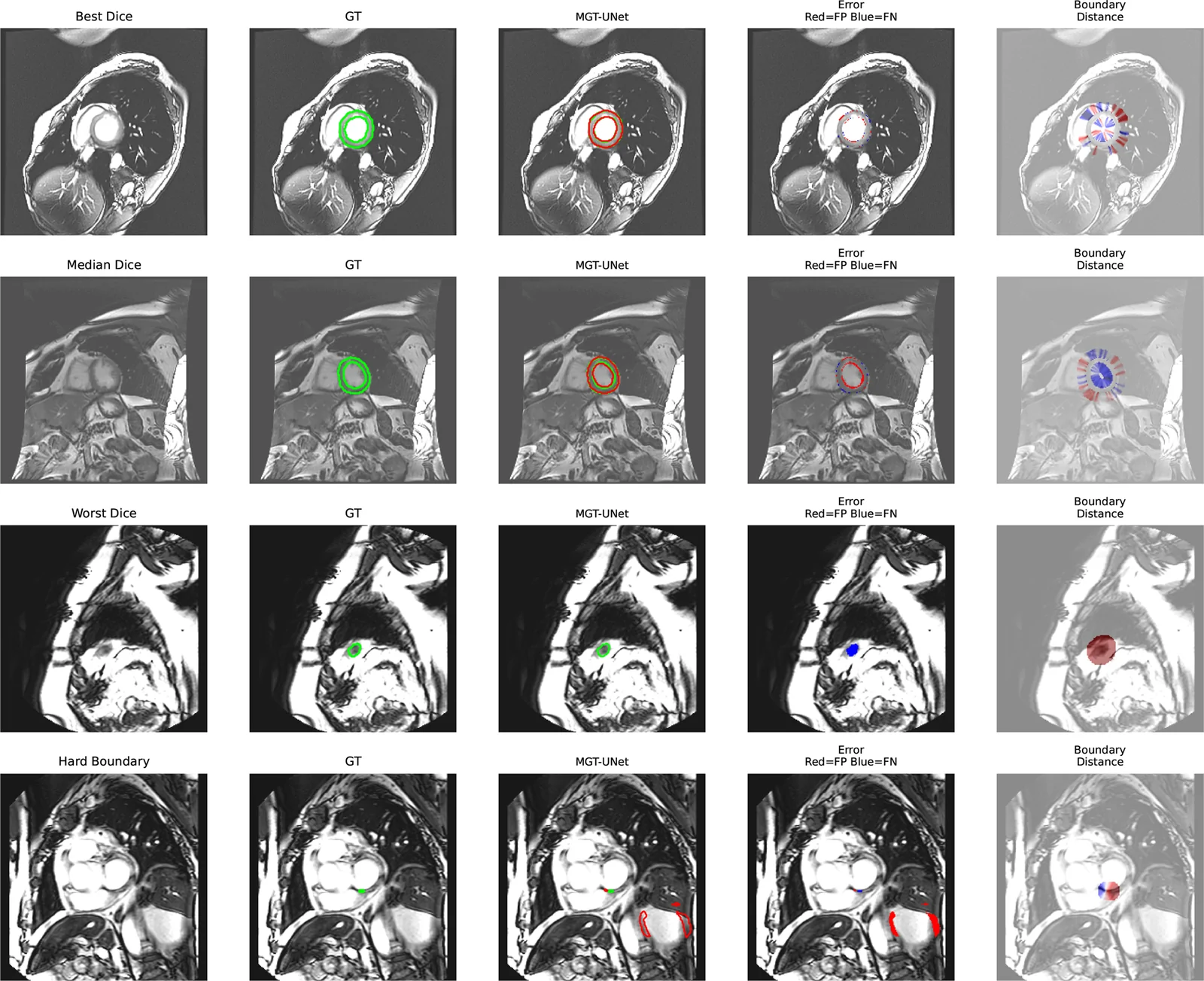
Qualitative segmentation results on the Synapse evaluation. Representative cases include the best, median, and worst Dice coefficients together with a hard-boundary example. Columns show the input image, GT, MGT–UNet prediction, error map, and signed boundary-distance visualization

To complement the quantitative evaluation, Figs. 8, 9, and 10 present qualitative segmentation results across the BUSI, ISIC2018, and Synapse datasets. These visualizations span a spectrum of performance levels (including best, median, and worst-case scenarios) alongside particularly challenging instances. Along with the predicted segmentation masks, we provide pixel-wise error maps and signed boundary-distance maps to explicitly illustrate localization errors and boundary alignment with the ground truth.

On the BUSI dataset (Fig. 8), MGT–UNet produces accurate lesion localization across representative cases, including images with weak boundaries, heterogeneous internal textures, and small lesions. In the best and median cases, the predicted contours closely follow the reference annotations, while most errors are confined to narrow boundary regions rather than large structural mismatches. In the hard-boundary example, both false-positive and false-negative regions appear near ambiguous lesion borders; however, the predicted mask preserves the overall lesion morphology with only localized deviations. Similarly, the small-lesion example shows that the model successfully localizes compact lesions while maintaining coherent boundaries. The signed boundary-distance maps further indicate that most boundary discrepancies are concentrated near lesion contours.

On the ISIC2018 and Synapse datasets (Figs. 9 and 10), the representative cases show that MGT–UNet generally preserves the target structures while most discrepancies remain localized near object boundaries. Compared with the BUSI examples, the qualitative differences among representative cases are less pronounced, reflecting the quantitative results presented in Sect. 5.1.

#### Probability map visualization

**Fig. 11:**
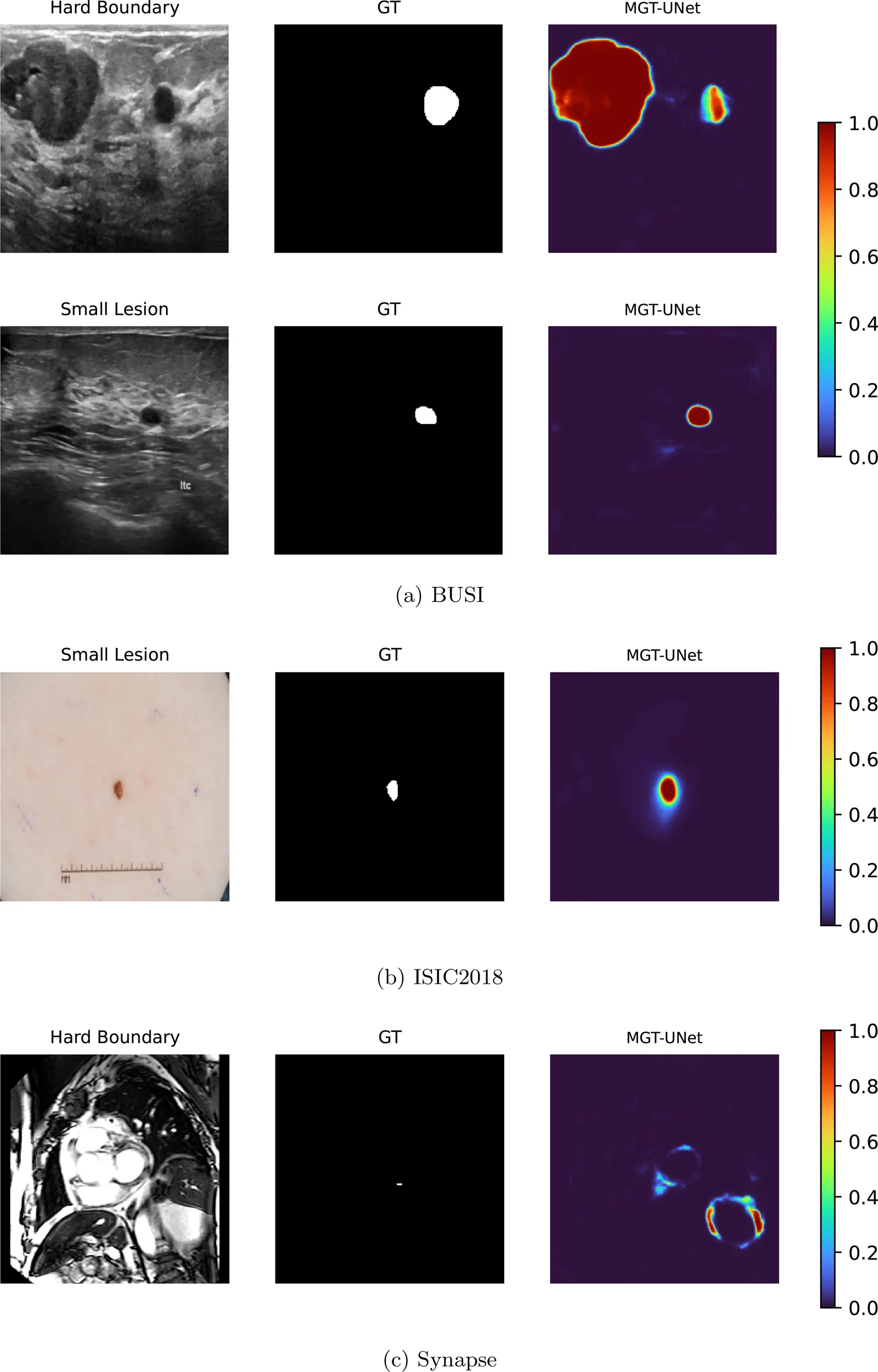
Representative probability maps generated by MGT–UNet on the BUSI, ISIC2018, and Synapse datasets. The heatmaps visualize the spatial confidence distribution of the predicted foreground regions

Figure 11 presents representative probability maps generated by MGT–UNet on the three evaluated datasets. The heatmaps provide a qualitative visualization of the spatial confidence associated with the predicted foreground regions.

On the BUSI dataset, high-confidence responses are concentrated within the lesion regions, with confidence gradually decreasing toward the lesion boundaries. In the hard-boundary example, uncertainty is primarily localized around ambiguous contour regions, whereas the lesion interior remains highly activated. The small-lesion example further shows that the model assigns concentrated confidence to compact lesions despite their limited spatial extent.

On the ISIC2018 and Synapse datasets, the representative probability maps illustrate the spatial confidence associated with the predicted segmentations. In both examples, regions of lower confidence are mainly located near object boundaries.

#### Boundary accuracy analysis

**Fig. 12:**
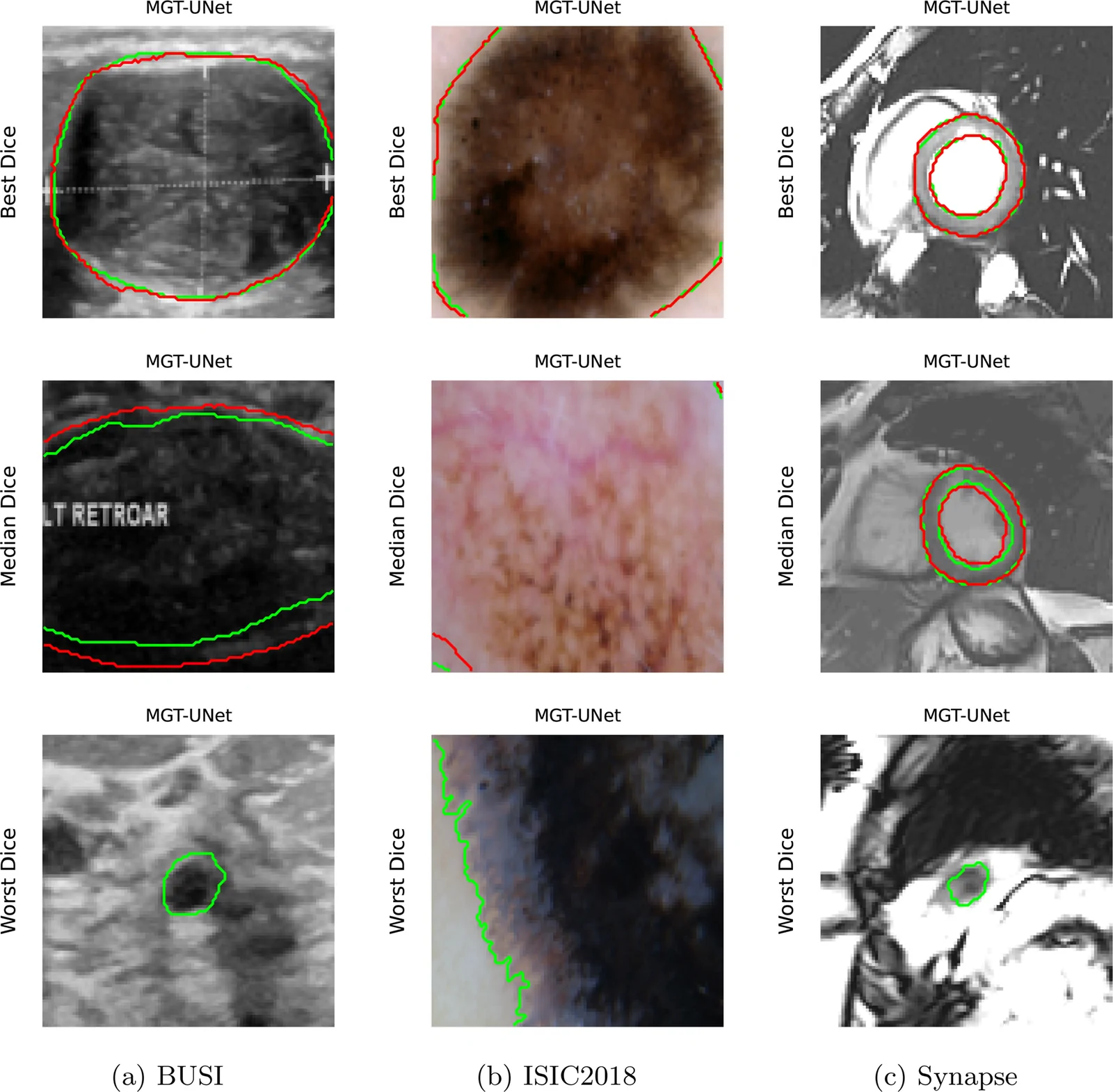
Zoomed comparisons of predicted and reference boundaries. Green contours denote the ground-truth annotation and red contours indicate the MGT–UNet prediction

Figure 12 presents enlarged views of representative boundary regions to facilitate visual assessment of boundary localization accuracy. The overlap between the predicted contour (red) and the reference annotation (green) provides a qualitative illustration of boundary agreement.

On the BUSI dataset, the best and median examples show close agreement between the predicted and reference contours. In the more challenging example, localized discrepancies are mainly observed near portions of the lesion boundary, while the overall lesion shape is preserved.

The representative examples from the ISIC2018 and Synapse datasets exhibit similar boundary comparisons. Most contour differences are localized to limited boundary regions, whereas the overall shapes of the predicted structures remain visually consistent with the reference annotations.

### Computational efficiency analysis

Table 9 summarizes the computational efficiency and training cost of the evaluated models on the BUSI dataset. The comparison includes the number of trainable parameters, GFLOPs, inference time, FPS, GPU memory consumption, the epoch achieving the best validation performance, and the corresponding training time.

**Table 9:** Computational efficiency and training cost comparison on the BUSI dataset. *↓* indicates lower values are preferred, while *↑* indicates higher values are preferred

Model	Params*↓* (M)	GFLOPs*↓*	t$_{infer}\downarrow$ (ms)	FPS*↑*	GPU*↓* (MB)	Epoch$_{best}\downarrow$	t$_{train}\downarrow$ (h)
U-Net	31.04	73.99	8.90	112.4	1813	52.0 *±* 25.5	0.34 *±* 0.06
DeepLabV3+	31.40	14.51	5.99	167.0	1022	67.0 *±* 1.4	0.17 *±* 0.03
UNet++	36.62	211.57	18.75	53.3	4104	64.0 *±* 11.3	0.95 *±* 0.07
TransUNet	105.28	50.72	12.64	79.1	2558	40.0 *±* 7.1	0.49 *±* 0.07
Swin-Unet	27.17	11.83	8.37	119.4	1241	63.5 *±* 46.0	0.34 *±* 0.18
BACFormer	93.78	23.54	33.72	29.7	7178	66.5 *±* 26.2	1.18 *±* 0.30
MGT–UNet (Ours)	237.89	364.66	25.95	38.5	11236	53.4 *±* 22.7	1.34 *±* 0.32

Note: The number of trainable parameters, GFLOPs, inference time (t_*infer*_), FPS, and GPU memory were measured using the final model checkpoint from a single representative run with identical hardware and software settings. The best epoch (Epoch_*best*_) and the training time (t_*train*_) are reported as mean ± standard deviation across independent training runs with different random seeds

Among the evaluated methods, MGT–UNet has the largest computational cost, with 237.89 M trainable parameters, 364.66 GFLOPs, and a peak GPU memory consumption of 11 236 MB. Its average inference time is 25.95 ms per image (38.5 FPS), and the best-performing checkpoint is obtained after *53.4 ± 22.7* epochs, corresponding to an average training time of *1.34 ± 0.32* h. In comparison, convolution-based models such as U-Net and DeepLabV3+ require substantially fewer computational resources, while transformer-based architectures generally occupy an intermediate position.

These results show that the improved segmentation performance of MGT–UNet on the BUSI dataset is accompanied by higher computational cost than the compared baseline methods in terms of model size, computational complexity, GPU memory usage, inference time, and training time.

### Ablation study

**Table 10:** Ablation configurations used to evaluate the principal components of MGT–UNet on the BUSI dataset

Model	TCE	GCT
Baseline		
M1 (M0 + TCE)	*\checkmark*	
M2 (M0 + GCT)		*\checkmark*
MGT–UNet (M0 + TCE + GCT)	*\checkmark*	*\checkmark*

To investigate the contribution of the principal architectural components, an ablation study was conducted on the BUSI dataset. The evaluated configurations are summarized in Table 11, and the corresponding quantitative results are presented in Table 12. Starting from the baseline encoder–decoder architecture, M1 incorporates the proposed TCE, M2 incorporates the GCT, and the complete MGT–UNet combines both components.

**Table 11:** Ablation study of the principal components of MGT–UNet on the BUSI breast ultrasound dataset. The baseline model is compared with variants incorporating the proposed TCE, the GCT, and their combination in the complete MGT–UNet architecture. Results are reported as mean *±* standard deviation over independent training runs. *↑* and *↓* indicate that higher and lower values are preferred, respectively

Model	Dice (%)*↑*	IoU (%)*↑*	HD$_{95}$*↓*	Precision (%)*↑*	Recall (%)*↑*
Baseline	72.59 *±* 2.90	65.91 *±* 3.23	23.90 *±* 2.83	74.56 *±* 4.42	75.21 *±* 2.48
M1	75.21 *±* 1.79	69.00 *±* 1.96	18.34 *±* 1.24	79.77 *±* 2.77	75.55 *±* 1.21
M2	75.33 *±* 1.70	69.21 *±* 1.72	16.94 *±* 0.69	77.96 *±* 2.85	76.50 *±* 1.80
MGT–UNet	77.74 *±* 1.41	71.24 *±* 1.39	18.11 *±* 1.36	80.35 *±* 1.58	79.16 *±* 1.50

**Table 12:** Software and hardware environment used for model training and evaluation

Component	Specification
Operating system	Ubuntu 22.04 LTS
Python	3.10
Deep learning framework	PyTorch
GPU	NVIDIA RTX A4000 (16 GB VRAM)
CUDA	CUDA-enabled GPU acceleration
CPU	Intel Core i9 Processor
System memory	32 GB RAM
Batch size	1
Input resolution	*224 × 224* pixels

Compared with the baseline, both M1 and M2 improve the overlap-based segmentation metrics. M1 increases the Dice from 72.59% to 75.21% while reducing HD$_{95}$ from 23.90 to 18.34. Similarly, M2 improves the Dice to 75.33% and achieves the lowest HD$_{95}$ (16.94) among the evaluated variants.

The complete MGT–UNet achieves the highest Dice (77.74%), IoU (71.24%), Precision (80.35%), and Recall (79.16%). Relative to the baseline, the complete model improves the Dice by 5.15 percentage points and the IoU by 5.33 percentage points. Although M2 achieves the lowest HD$_{95}$, the complete model provides the best overall balance across the evaluated segmentation metrics.

## Discussion

This study presented MGT–UNet, a hybrid CNN–Transformer architecture that combines multi-scale convolutional feature extraction with bottleneck-level global contextual modeling for breast ultrasound segmentation. The experimental results demonstrate that the proposed architecture is most effective on the BUSI dataset, where it achieves the highest overall segmentation performance among the evaluated methods. The additional evaluations on the ISIC2018 and Synapse datasets further provide insight into the behavior of the proposed architecture under different imaging modalities.

The performance gains observed on the BUSI dataset can be attributed to the complementary roles of the proposed architectural components. Breast ultrasound images are often affected by speckle noise, weak contrast, and irregular lesion boundaries, making both local feature representation and global contextual understanding important for accurate segmentation. The TCE captures image features at multiple receptive fields, whereas the GCT incorporates long-range contextual information at the bottleneck stage. The ablation study indicates that each component individually improves segmentation performance relative to the baseline, while their combination yields the highest overall Dice and IoU, suggesting that local multi-scale feature learning and global contextual modeling complement one another.

The qualitative analysis provides additional support for the quantitative findings. On representative BUSI cases, the predicted contours generally align well with the reference annotations, and the remaining discrepancies are mainly localized near lesion boundaries. The probability maps demonstrate high prediction confidence concentrated within lesion regions, while boundary visualizations confirm that most discrepancies are confined to ambiguous contour areas. Together, these observations are consistent with the improved overlap-based metrics and reduced HD$_{95}$ achieved on the BUSI dataset.

The additional experiments on the ISIC2018 and Synapse datasets indicate that the proposed architecture can be applied to other binary medical image segmentation tasks, although the performance improvements are less pronounced than those observed on BUSI. These findings suggest that the principal benefit of MGT–UNet is most evident for breast ultrasound segmentation, which is the primary application considered in this study.

The computational efficiency analysis shows that the improved segmentation performance is accompanied by increased computational cost. Compared with the evaluated baseline models, MGT–UNet requires more parameters, higher computational complexity, greater GPU memory usage, and longer inference and training times. This increase reflects the additional architectural components introduced to enhance feature representation and contextual modeling, highlighting the trade-off between segmentation accuracy and computational efficiency.

Several limitations should be acknowledged. First, the experimental evaluation focuses primarily on the BUSI dataset, while the ISIC2018 and Synapse experiments serve as additional evaluations rather than comprehensive benchmarks across imaging modalities. Second, although MGT–UNet achieves the highest mean Dice on BUSI, the image-level statistical analysis does not identify a statistically significant difference from the strongest baseline, indicating that further validation on larger cohorts or additional datasets is warranted. Third, the current implementation is limited to two-dimensional image segmentation and has not been evaluated on volumetric three-dimensional data. Finally, the increased computational requirements may limit deployment in resource-constrained environments.

Future work will extend MGT–UNet to standard multi-class and three-dimensional medical image segmentation tasks and evaluate the proposed architecture on larger and more diverse clinical datasets. Further investigation of lightweight multi-scale feature extraction and more efficient transformer designs may reduce computational cost while preserving segmentation accuracy. In addition, self-supervised and semi-supervised learning strategies will be explored to improve robustness when annotated training data are limited.

## Conclusion

This paper introduced MGT–UNet, a hybrid CNN–Transformer architecture for breast ultrasound image segmentation. The proposed architecture integrates a TCE and a GCT within a unified encoder–decoder framework to combine multi-scale local feature extraction with bottleneck-level global contextual modeling.

Experimental results show that MGT–UNet achieves the best overall segmentation performance on the BUSI dataset among the evaluated methods. The ablation study indicates that combining the TCE and GCT yields higher segmentation performance than using either component individually. Additional experiments on the ISIC2018 and Synapse datasets further illustrate the behavior of the proposed architecture under different imaging modalities.

The improved segmentation performance is accompanied by increased computational cost, indicating a trade-off between segmentation performance and computational efficiency. Future work will investigate lightweight multi-scale feature extraction modules and more efficient self-attention mechanisms to reduce computational complexity. In addition, the proposed architecture will be extended to standard multi-class and three-dimensional medical image segmentation tasks and evaluated on larger, multi-center clinical datasets.

## Figures and Tables

**Fig. 13:**
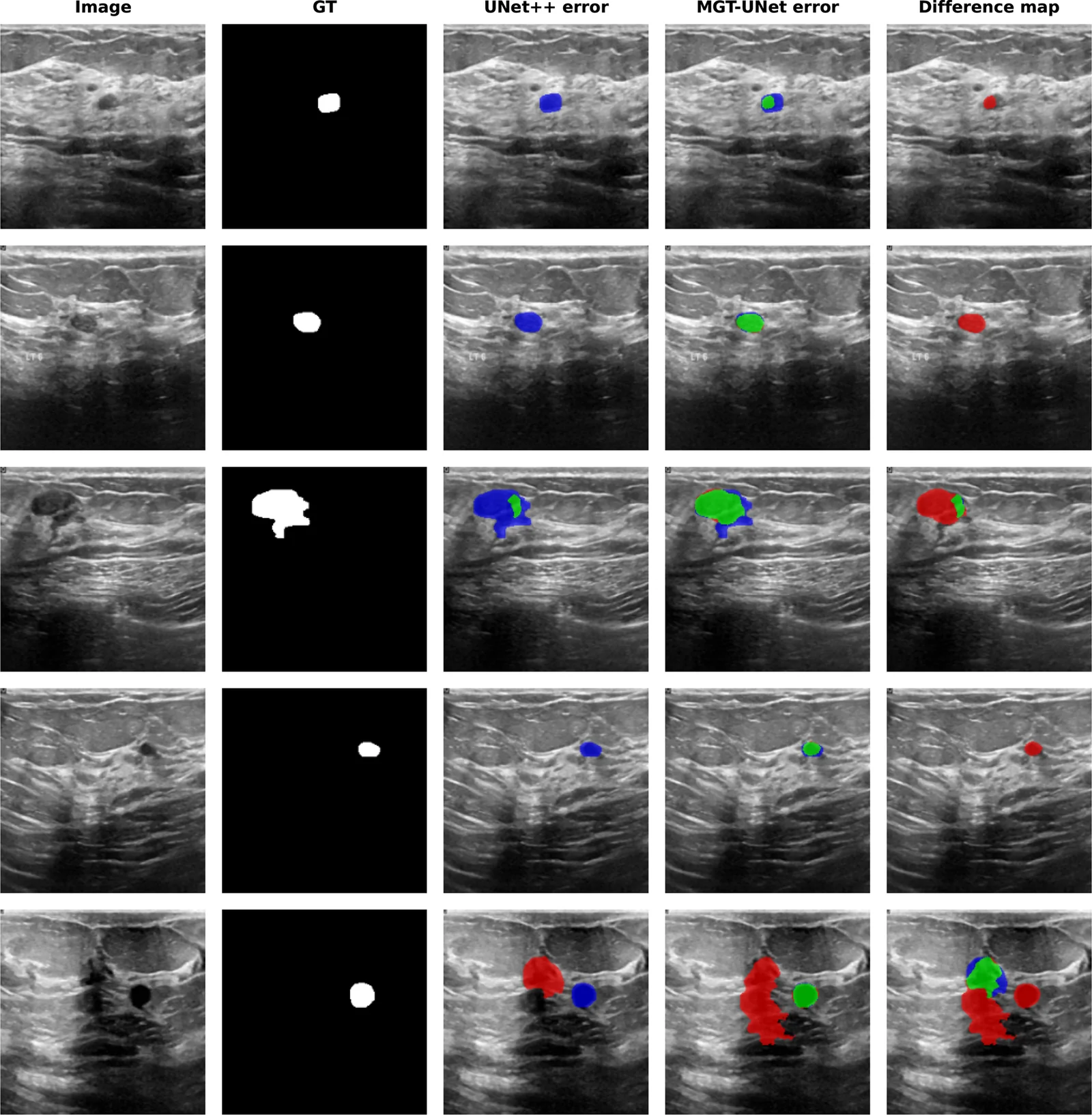
Representative cases in which MGT–UNet outperformed UNet++. Compared with UNet++, MGT–UNet consistently recovers lesion regions that are partially or completely missed by the baseline, resulting in substantial improvements in Dice. The examples include both small lesions and lesions with irregular morphology or weak boundaries, illustrating the robustness of the proposed architecture across different lesion characteristics

**Fig. 14:**
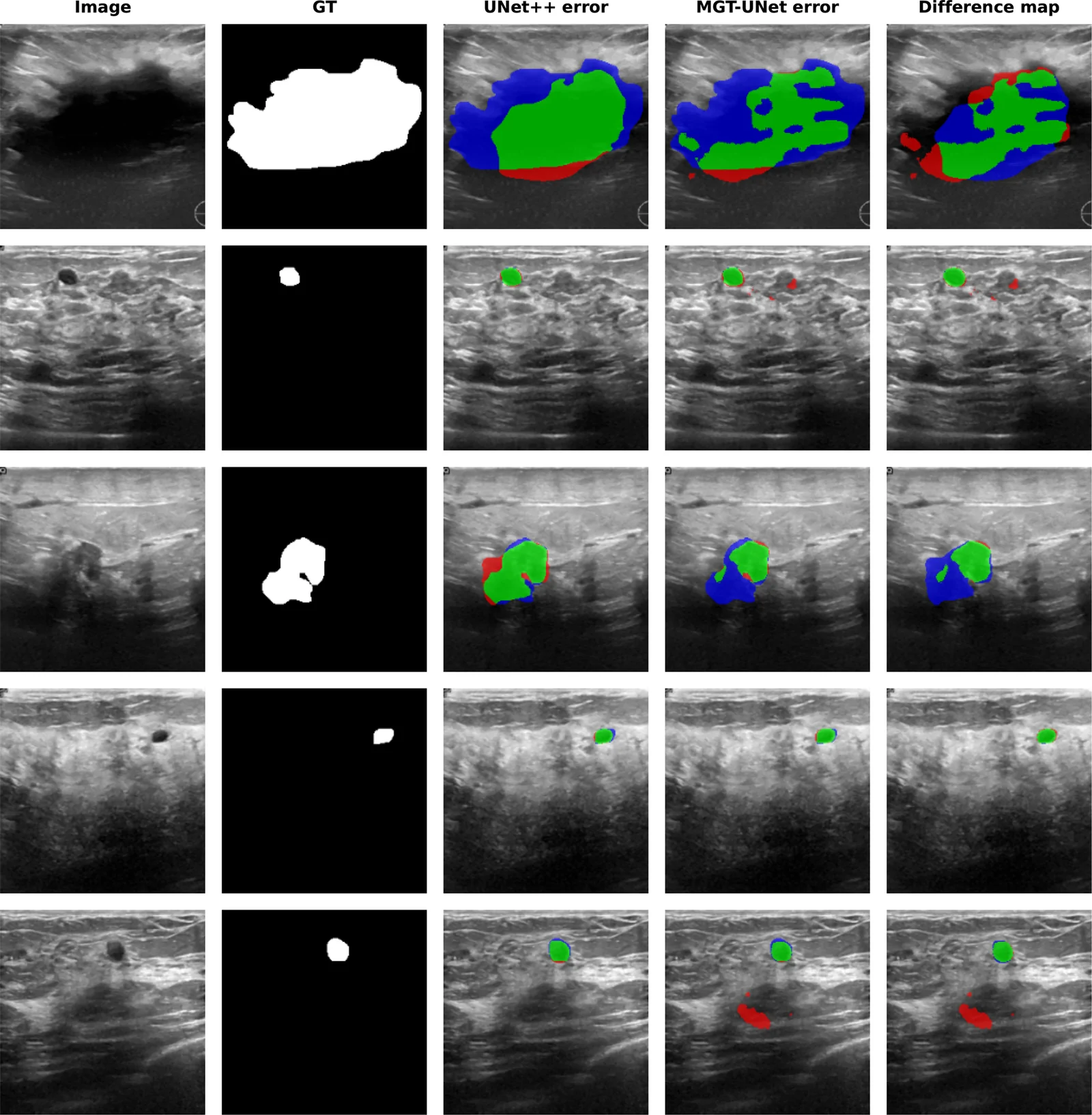
Representative cases in which UNet++ outperformed MGT–UNet. These examples show that MGT–UNet may introduce additional false-positive regions or slightly less accurate lesion boundaries in some challenging images. Nevertheless, the predicted lesion generally maintains substantial overlap with the ground truth, indicating that the degradation is more frequently associated with over-segmentation or boundary imprecision than complete lesion omission

## Data Availability

The datasets used in this study are publicly available at the following sources: BUSI dataset: https://scholar.cu.edu.eg/?q=afahmy/pages/dataset. ISIC2018 dataset: https://challenge.isic-archive.com/data/#2018. Synapse dataset: https://www.synapse.org/#!Synapse:syn3193805.

## A Software environment and implementation details

To facilitate reproducibility, this appendix summarizes the software environment and implementation details used throughout all experiments. Unless otherwise specified, the proposed MGT–UNet and all baseline methods were implemented and evaluated under the same hardware and software environment.

The software environment was managed using a dedicated Python virtual environment to ensure consistent dependency versions throughout model development, training, and evaluation. The same implementation environment was used for all compared methods to guarantee that performance differences arose from architectural design rather than software configuration.

The implementation relies on several widely adopted open-source scientific computing and medical image analysis libraries. Tables 13 and 13 summarizes the principal software dependencies.

**Table 13:** Principal software libraries used in the implementation

Library	Purpose
PyTorch	Deep learning implementation
TorchVision	Neural network utilities and pretrained models
OpenCV	Image processing and I/O
Albumentations	Data preprocessing and augmentation
scikit-image	Image processing utilities
Nibabel	Medical image input/output
MedPy	Medical image evaluation metrics
Matplotlib	Visualization
Pandas	Data management and analysis
NumPy	Numerical computation

The principal software dependencies are also listed in the accompanying requirements.txt file, including OpenCV, Albumentations, scikit-image, nibabel, matplotlib, MedPy, and pandas. Throughout all experiments, identical preprocessing pipelines, optimization strategies, dataset partitions, and evaluation protocols were employed for the proposed method and all baseline models. This unified experimental environment ensures a fair and reproducible comparison of segmentation performance and computational efficiency.

## B Representative paired-case analysis

Figures 13 and 14 present representative lesion-present BUSI cases selected according to the largest positive and negative mean paired Dice differences averaged across five independent training runs. Each row contains the input image, the ground-truth mask, the error maps of UNet++ and MGT–UNet, and the prediction-difference map. In the error maps, green denotes true-positive pixels, red denotes false-positive pixels, and blue denotes false-negative pixels. In the difference maps, red indicates pixels predicted only by MGT–UNet, blue indicates pixels predicted only by UNet++, and green indicates pixels correctly predicted by both models.

The representative winning cases shown in Fig. 13 reveal several characteristic improvements achieved by MGT–UNet. First, compared with UNet++, MGT–UNet consistently reduces false-negative regions, as evidenced by the noticeably smaller blue regions in the MGT–UNet error maps. In several cases, lesion regions that are only partially detected or completely missed by UNet++ are successfully recovered by MGT–UNet, leading to substantial improvements in Dice. Second, MGT–UNet produces more complete lesion coverage for lesions exhibiting irregular morphology or weakly defined boundaries, resulting in larger overlap with the ground truth. Third, the prediction-difference maps demonstrate that most additional regions predicted by MGT–UNet correspond to true lesion pixels rather than isolated false-positive regions, indicating that the performance gains primarily arise from improved lesion recovery instead of indiscriminate expansion of the segmentation mask.

The representative losing cases in Fig. 14 provide complementary insight into the limitations of the proposed model. In these examples, MGT–UNet generally remains capable of detecting the target lesion but occasionally introduces additional false-positive regions or less precise lesion boundaries than UNet++. Importantly, complete lesion omission is uncommon in these representative cases; instead, the performance degradation is mainly attributable to over-segmentation or boundary leakage. These observations indicate that MGT–UNet tends to preserve lesion sensitivity while sacrificing a modest amount of boundary precision in some challenging images.

A notable observation is the asymmetric error characteristics between the winning and losing cases. The improvements achieved by MGT–UNet are predominantly associated with reducing false-negative regions, whereas its degradations are more frequently caused by increased false-positive regions or slight boundary leakage. This asymmetry is consistent with the statistical analysis presented in Sect. 4.6, where MGT–UNet achieves a higher mean Dice score despite the non-significant Wilcoxon signed-rank test. Collectively, the qualitative analysis suggests that the proposed architecture is particularly effective at recovering difficult lesion regions that are overlooked by UNet++, while its remaining errors are primarily related to boundary refinement rather than failure to detect the lesion itself.

## Code availability

The implementation of MGT–UNet will be made publicly available upon acceptance.
